# Do Obesity Classifications Create the Obesity Paradox? A Scoping Review of Obesity Definitions Applied in Sepsis Research

**DOI:** 10.1111/cob.70039

**Published:** 2025-08-08

**Authors:** Efris Kartikasari, Brian Robinson, Caz Hales

**Affiliations:** ^1^ School of Nursing, Midwifery, and Health Practice Victoria University of Wellington Wellington New Zealand

**Keywords:** clinical outcomes, obesity, obesity definitions, sepsis

## Abstract

Obesity appears to be associated with improved health outcomes in patients with sepsis, a phenomenon termed the obesity paradox. However, the potential influence of varying operational definitions of obesity on clinical outcomes within this paradox remains inadequately characterised. This scoping review aimed to identify, analyse, and synthesise the methodological approaches to obesity definition employed in sepsis research. A systematic literature search was conducted in August 2023 across MEDLINE, Embase, CINAHL, and CENTRAL databases. This review included original articles, systematic reviews, and meta‐analyses reporting on adult patients with both obesity and sepsis. After removing 60 duplicates, 430 citations were screened, and 68 met the inclusion criteria. Among studies on the obesity paradox, 90.5% supporting and 88.6% refuting it employed body mass index‐based definitions, with approximately three‐quarters using retrospective designs. Studies supporting the obesity paradox identified patients with obesity as younger, predominantly female, and with higher comorbidity rates. In contrast, studies refuting the paradox reported more diverse age and sex distributions, yet consistently noted elevated chronic disease prevalence in patients with obesity. Both groups found similar or higher illness severity scores among patients with obesity. The lack of methodological rigour in obesity definitions within clinical research may contribute to the obesity paradox. Future studies should critically evaluate measurement methods and definitional variability to clarify their impact on clinical outcomes.

**Summary**

- What is already known about this subject
  ○ The obesity paradox, where patients with obesity demonstrate superior clinical outcomes compared to those with a BMI in the healthy range, has been documented in patients with sepsis.
  ○ Body mass index (BMI), the predominant anthropometric measure utilised in clinical settings, exhibits methodological limitations in differentiating between adipose tissue, skeletal muscle, and bone mineral density.
  ○ The relationship between varying operational definitions of obesity and patient outcomes remains inadequately characterised in the existing literature.
- What this study adds
  ○ Most studies investigating the relationship between obesity and clinical outcomes, whether supporting or refuting the obesity paradox, predominantly employed BMI‐based anthropometric classification systems.
  ○ Methodological considerations, particularly the operational definition of obesity, demonstrate significant potential to influence research findings and subsequent clinical interpretations.
  ○ Future research should apply explicit, consistent operational definitions of obesity when examining the relationships between adiposity and clinical outcomes in patient populations.

## 1 Introduction

Interest in the impact of obesity on sepsis, a critical condition due to a dysregulated body response to an infection [1], has increased in parallel with the global rise in obesity prevalence. According to the World Health Organisation (WHO), over 890 million adults were living with obesity in 2022, with the worldwide prevalence of obesity more than doubling since 1990 [2]. Notably, some studies delving into the association between obesity and health outcomes have found that obesity is linked to reduced mortality in patients with sepsis [3, 4, 5, 6, 7]. For instance, a study involving 3145 patients with sepsis found that patients with obesity, diabetes, and hypertension had a lower risk of sepsis‐related mortality compared to those with a body mass index (BMI) in the healthy range [7]. Additionally, a meta‐analysis of 15 observational studies that used BMI as a measure of body size reported that overweight and obesity were associated with reduced mortality within 30 days among patients with sepsis [3].

The obesity paradox is a clinical hypothesis suggesting that, in specific patient populations, obesity may confer a survival advantage [8]. Robinson et al. [9] postulated a conceptual model in which inflammation serves as a link between obesity and sepsis. In individuals with obesity, chronic low‐grade inflammation attenuates the body's response during the acute inflammatory state of sepsis, resulting in a mild increase in cytokines. This moderated response appears to reduce tissue damage and organ dysfunction, potentially contributing to better survival outcomes compared to patients with a BMI in the healthy range [9].

Conflicting perspectives have emerged regarding the paradoxical association between obesity and improved clinical outcomes in patients with sepsis. Sceptics of the obesity paradox contend that methodological factors may underlie this phenomenon, particularly misclassification bias stemming from imprecise body size measurements and inadequate control for confounding variables [10, 11]. Examination of literature supporting the obesity paradox has highlighted several methodological limitations, including the predominance of retrospective study designs, inherent constraints of BMI as a measure of adiposity distribution, and insufficient accounting for the complex pathophysiological mechanisms associated with obesity [12, 13].

Recently, a consortium of 58 experts proposed a diagnostic framework that distinguishes between preclinical and clinical obesity based on the presence of obesity‐related organ dysfunction and limitations in activities of daily living [14]. Exclusive reliance on BMI‐based obesity classification systems may result in diagnostic imprecision, as such approaches fail to incorporate assessments of organ functionality or individual capacity to perform activities in daily living; both represent fundamental parameters in comprehensive health assessment [14]. This methodological issue raises a critical epistemological question: To what extent do the operational definitions of obesity used in clinical research, particularly those predicated solely on BMI criteria, influence observed health outcomes?

To our knowledge, no prior study has specifically examined the methodological approaches to obesity operationalisation, despite the critical importance of accurate adiposity assessment in clinical research. Accordingly, this scoping review aims to identify, analyse, and critically evaluate existing obesity measurement methodologies employed in studies investigating the relationship between obesity and clinical outcomes among patients with sepsis.

## 2 Methods

### 2.1 Study Design

This scoping review was conducted in accordance with the Joanna Briggs Institute framework, initially proposed by Arksey and O'Malley in 2005 and later enhanced by Levac et al. in 2010 and Peters et al. in 2020 [15]. The review protocol was registered with the Open Science Framework under the registration DOI https://doi.org/10.17605/OSF.IO/Q7KMD.

### 2.2 Identifying the Research Question

This scoping review addressed the following questions: (i) How is obesity defined in clinical studies? (ii) What are the characteristics of patients with obesity who were included in these studies? (iii) What research methodologies are employed in studies supporting the obesity paradox? (iv) What are the strengths, limitations, and implications of the obesity definitions used, and what recommendations can be made for future research?

### 2.3 Search Strategy

The search strategy was developed in collaboration with a health subject librarian. Details of the search strategy are presented in Table 1. MEDLINE (Ovid), Embase (Ovid), CINAHL, and CENTRAL were systematically searched to identify relevant titles and abstracts. Searches were conducted in August 2023 and were restricted to English‐language articles published between January 2012 and December 2023. The 10‐year timeframe was chosen to pragmatically focus the review on contemporary studies examining how obesity is defined in clinical research.

**TABLE 1:** Search terms.

Database	Key terms
MEDLINE	exp Obesity, Morbid/or exp. Obesity Paradox/or exp. Obesity, Abdominal/ exp. Sepsis/ 1 and 2
Embase	*morbid obesity/or *metabolically benign obesity/or *abdominal obesity/or *sarcopenic obesity/or *obesity paradox/ Exp sepsis/ 1 and 2
CINAHL	S1 (MH “Obesity+) OR (MM “Obesity Paradox”) OR (MM “Obesity, Morbid”) S2 (MH “Sepsis+) S3 S1 AND S2
CENTRAL	(obesity OR “obesity paradox” OR “morbid obesity”) AND (sepsis OR septic) in Title Abstract Keyword

### 2.4 Eligibility Criteria

The inclusion criteria for this scoping review were studies that enrolled adult patients (aged 18 years or older) with both obesity and sepsis in hospital settings. Pregnant women were excluded due to physiological changes associated with pregnancy, such as gestational weight gain, which is typically temporary and does not reflect standard patterns of obesity [16].

This scoping review included publications from 1 January 2012 to 31 December 2023, encompassing original research articles, systematic reviews, and meta‐analyses. We considered quantitative study designs, including randomised and non‐randomised controlled trials, prospective and retrospective cohort studies, cross‐sectional studies, case–control studies, and case series. Secondary analyses of quantitative studies and Mendelian randomisation studies reporting relevant health outcomes were also eligible. Letters to the editor were included only if they provided sufficient information about the study and were not solely intended as commentary or critique. We excluded qualitative studies, clinical guidelines, conference abstracts, opinion papers, and letters lacking sufficient methodological detail.

### 2.5 Screening and Data Extraction

Following the search, all retrieved articles were imported into Covidence (version 2.0; Veritas Health Innovation, Melbourne, Australia) for screening and data management. Duplicate articles were identified and removed. Authors E.K. and C.H. then screened the remaining articles for suitability based on the inclusion and exclusion criteria. Any disagreements between E.K. and C.H. were resolved through discussion with B.R. As a result, eligible articles were selected for full‐text review, and the reasons for excluding sources were documented and displayed in the PRISMA diagram. Following this, a full‐text analysis was independently performed by E.K., C.H., and B.R., compliant with the inclusion criteria. Discrepancies were resolved through discussion and consensus among all reviewers.

A data extraction table was developed in Microsoft Excel, following the data charting framework proposed by Arksey and O'Malley [17]. The table was piloted using three studies and reviewed by two authors (E.K. and CH) to confirm its suitability and comprehensiveness in recording data relevant to the review questions, as recommended by Peters et al. [15] Information on patients' length of stay, severity, and mortality was recorded to address the research objectives. These outcome measures will help determine whether the study identifies an obesity paradox among studies. After EK extracted the data, CH and BR reviewed a randomly selected 10% of the data to ensure accuracy.

### 2.6 Data Presentation

Extracted data are presented in both tabular and graphical formats, accompanied by narrative summaries that describe findings relevant to the objectives of this scoping review. To provide structured information, the results of this scoping review are organised based on obesity definitions, study populations, research methodologies, and health outcomes. Infographics summarising key study characteristics were generated from numerical analysis of the included studies.

## 3 Results

### 3.1 Literature Search Results

**FIGURE 1:**
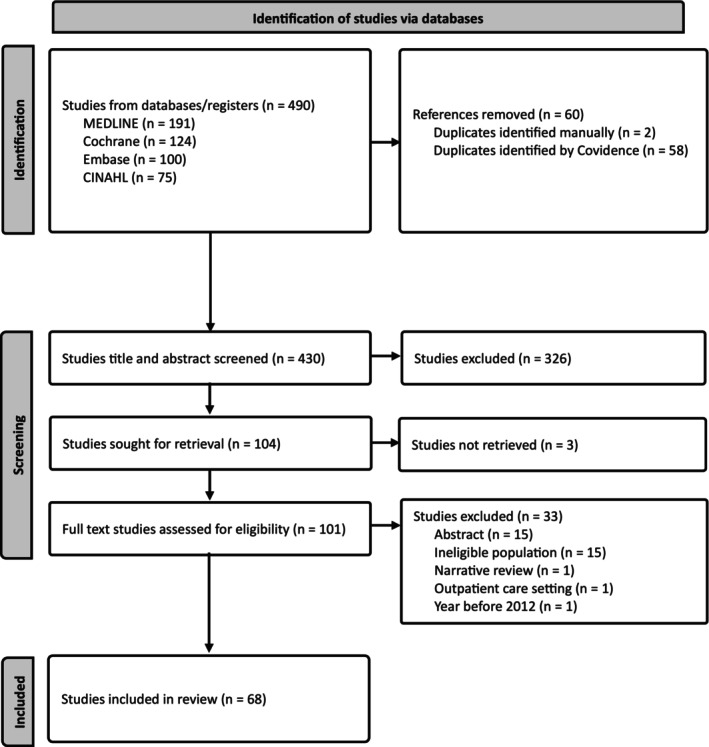
PRISMA flow chart depicting the search strategy and selection of studies.

The literature search yielded 490 articles. After removing 60 duplicates, 430 citations were screened. Title and abstract screening narrowed the selection to 104 studies, of which three could not be retrieved, leaving 101 studies for full‐text review. We included 68 studies that met the inclusion criteria for this review. The search results and screening process are shown in Figure 1.

### 3.2 Study Descriptions

Among the included studies, the most common design was retrospective (e.g., retrospective cohort, retrospective chart review, case–control, and retrospective database studies), used in 49 studies (72%). Prospective designs (e.g., prospective cohort and prospective comparative studies) accounted for 10 studies (14.8%). The other nine studies (13.2%) consisted of systematic review, meta‐analysis, Mendelian randomisation study, and secondary analysis study. Study designs are depicted in Figure S1.

Nearly half of the studies (48.5%) were located in the United States (U.S.), with Canada contributing four studies (5.9%). The remaining studies originated from Europe (15; 22.1%), West Asia (7; 10.3%), East Asia (6; 8.8%), Australia (2; 2.9%), and South America (1; 1.5%). This distribution may reflect that obesity and sepsis are significant health concerns in the U.S. as more than 100 million U.S. adults are living with obesity, and over 22 million are living with severe obesity between 2017 and March 2020 [18].

### 3.3 The Definitions of Obesity in Clinical Studies of Sepsis

Figure S2 displays the obesity measurement methods used in the included studies. The majority of studies (60; 88.2%) defined obesity solely based on BMI, calculated by dividing body weight in kilograms by height in meters squared (kg/m^2^). Only eight studies (11.8%) used other measures [19, 20, 21, 22, 23, 24, 25, 26], such as visceral adipose tissue to subcutaneous adipose tissue ratio (VAT/SAT) [22], visceral and sarcopenic obesity [21], the International Classification of Diseases, Ninth Revision (ICD‐9) codes for obesity [19, 23, 24], and the combination of BMI with other metrics [20, 25, 26].

The use of BMI to measure obesity was aligned with guidelines from the World Health Organisation (WHO), the Centres for Disease Control and Prevention (CDC), the National Institutes of Health (NIH), and the National Heart, Lung, and Blood Institute (NHLBI). Generally, individuals were categorised as having underweight (BMI < 18.5 kg/m^2^), a BMI in the healthy range (BMI 18.5–24.9 kg/m^2^), overweight (BMI 25–29.9 kg/m^2^), and obesity (BMI ≥ 30 kg/m^2^). Some authors further stratified obesity into Class I (BMI 30–34.9 kg/m^2^), Class II (BMI 35–39.9 kg/m^2^), and Class III (BMI ≥ 40 kg/m^2^, referred to as severe obesity) [27, 28, 29, 30, 31]. Severe obesity was also defined as a BMI greater than or equal to 35 kg/m^2^ in the presence of obesity‐related comorbidities such as hypertension, congestive heart failure, and diabetes [32, 33]. However, 19% (*n* = 13) of the studies collapsed Class 1 and Class 2 obesity into a single category (BMI = 30.0 to 39.99 kg/m^2^) [26, 34, 35, 36, 37, 38, 39, 40, 41, 42, 43, 44, 45]. Additionally, three studies further classified obesity using two cut‐offs: obesity (BMI 30.0–34.9 kg/m^2^) and severe obesity (BMI ≥ 35 kg/m^2^) [46, 47, 48].

Among studies examining clinical indicators such as the risk of sepsis, illness severity, length of stay (LOS), or mortality, two major discourses on the obesity paradox were identified. Studies supporting the paradox concluded better outcomes for patients with obesity compared to those with a BMI in the healthy range, whereas studies refuting the paradox found similar or worse outcomes. Of the 68 articles, 20 original studies and 1 meta‐analysis supported the paradox, while 35 studies refuted it. Two systematic reviews reported mixed findings, either supporting or refuting the obesity paradox [25, 44]. Five of the seven studies [34, 39, 48, 49, 50] included in the systematic review by Trivedi et al. [44] and nine research studies [19, 24, 34, 36, 39, 42, 48, 49, 50] from the systematic review by Robinson et al. [25] are part of this scoping review. The remaining 10 studies were categorised as “not applicable” as they focused on pharmacodynamics or pharmacokinetics, assessed the predictive value of obesity measures for adverse outcomes, or analysed cohorts composed exclusively of patients with obesity [20, 32, 51, 52, 53, 54, 55, 56, 57, 58].

### 3.4 Description of Studies Supporting and Refuting the Obesity Paradox

#### 3.4.1 Methodology

A summary of studies investigating the obesity paradox in patients with sepsis is shown in Table 2. Among studies supporting the obesity paradox, 17 were retrospective cohort studies, 2 were prospective cohort studies, 1 was a secondary analysis, and 1 was a meta‐analysis. Of the 13 studies included in the meta‐analysis, 5 research studies [30, 34, 36, 48, 60] are covered in this review. Likewise, of studies refuting the obesity paradox, 26 employed retrospective designs. The remaining studies employed various designs, including prospective studies (*n* = 4), a secondary analysis of a prospective cohort study (*n* = 1), a correlational study (*n* = 1), a case–control study (*n* = 1) and Mendelian randomisation studies (*n* = 2).

**TABLE 2:** Studies investigating the obesity paradox in patients with sepsis.

Author, year, country	Study design	Obesity classification	Sample size	Age (years), mean ± SD or median (range)	Male (%)	Main findings
Studies supporting the obesity paradox						
Sakr et al. [59] 2012, Italy	Retrospective cohort study	BMI	3920	UW = 63.2 ± 19.6 NW = 63.4 ± 17.7 OW = 64.5 ± 15.1 OB = 66.1 ± 12.9 SO = 62.2 ± 13.5	UW = 48 NW = 55 OW = 74.2 OB = 58.1 SO = 38.9	Patients with overweight and obesity had a lower risk of ICU death.
Kuperman et al. [39] 2013, USA	Retrospective cohort study	BMI	792	UW = 63.2 ± 19.6 NW = 63.4 ± 17.7 OW = 64.5 ± 15.1 OB = 66.1 ± 12.9 SO = 62.2 ± 13.5	UW = 34.7 NW = 59.4 OW = 57.8 OB = 54.5 SO = 26.1	Severe obesity appeared to confer a protective effect against inpatient sepsis mortality. Survivors had higher BMI compared with non‐survivors.
Wacharasint et al. [50] 2013, Canada	Retrospective cohort study	BMI	730	BMI < 25 = 63 (48–74) OW = 64 (50–73) OB = 63 (53–72)	BMI < 25 = 63 OW = 67.9 OB = 55.5	Compared with patients with a BMI of less than 25 kg/m^2^, patients with overweight and obesity had a lower 28‐day mortality.
Prescott et al. [48] 2014, USA	Retrospective cohort study	BMI	1404	NW = 81.3 ± 8.6 OW = 78.7 ± 8.6 OB = 75.3 ± 8.1 SO = 72.8 ± 8.0	NW = 46.6 OW = 57.9 OB = 46 SO = 28	Patients with obesity and severe obesity had lower odds of 90‐day mortality and a reduced risk of 1‐year mortality compared to those with a BMI in the healthy range.
Abbate et al. [19] 2016, USA	Secondary analysis of study	ICD‐9‐based chronic disease indicator	116 566	NR	NR	Obesity was inversely associated with in‐hospital mortality. Obesity was associated with lower mortality in adults aged 50 and older, but not in those under 50.
Nguyen et al. [24] 2016, USA	Retrospective cohort study	ICD‐9‐CM codes	1 763 000	NO = 70 (56–81) OB = 62 (52–71)	NO = 50.9 OB = 40.3	Obesity was associated with a 16% reduction in mortality.
Zhou et al. [60] 2018, China	Retrospective cohort study	BMI	178	UW = 79 (69–86) NW = 78 (67–84) OW = 73 (57–83) OB = 77 (71–86)	UW = 24 NW = 70 OW = 20 OB = 2	Higher BMI was associated with improved prognosis.
Pepper et al. [61] 2019, USA	Retrospective cohort study	BMI	55 038	UW = 71 (58–83) NW = 73 (59–84) OW = 71 (58–82) OB class I = 68 (56–77) OB class II = 65 (55–75) OB class III = 61 (51–70)	UW = 43.8 NW = 51.8 OW = 55.5 OB class I = 51.1 OB class II = 44.3 OB class III = 36.2	Compared to patients with a BMI in the healthy range, short‐term mortality was lower in those with overweight and obesity.
Jagan et al. [28] 2020, USA	Retrospective cohort study	BMI	7967	UW = 69 (57–81) NW = 71 (56–83) OW = 69 (56–80) OB = 64 (53–74)	UW = 43.9 NW = 50.7 OW = 54.3 OB = 47	Unadjusted in‐hospital mortality rate was lower for higher BMI categories. The obesity paradox was evident only in patients with mean arterial pressure ≥ 65 mm.
Kotecha et al. [62] 2020, USA	Retrospective cohort study	BMI	2017	UW = 62.4 (50.5–71.7) NW = 65.3 (54.1–76.1) SO = 61.7 (53.2–69.6)	UW = 43.5 NW = 55.8 SO = 53.9	In‐hospital mortality decreased with higher BMI.
Lin et al. [63] 2020, Israel	Retrospective cohort study	BMI	7967	UW = 67.02 ± 17.6 NW = 67.56 ± 16.88 OW = 66.51 ± 16.21 OB = 63.71 ± 14.36	UW = 47.69 NW = 54.24 OW = 60.28 OB = 52.48	Patients with obesity had a lower risk of ICU mortality and 28‐day mortality compared to those with a BMI in the healthy range.
Shimada et al. [64] 2020, Canada	Retrospective cohort study	BMI	519	NR	NR	Twenty‐eight‐day survival increased with the number of minor rs7852409 C alleles (VLDLR GOF allele), from 0 (GG) to 1 (GC) to 2 (CC). Patients with obesity have more adipose tissue and therefore a higher total number of adipocytes VLDLR.
Alsio et al. [46] 2021, Sweden	Prospective cohort study	BMI	1656	UW = 68 ± 22 NW = 68 ± 20 OW = 69 ± 16 OB = 66 ± 16 SO = 62 ± 14	UW = 38.9 NW = 55.5 OW = 60.6 OB = 51 SO = 47.6	Higher BMI was associated with lower 28‐day and one‐year case fatality rate.
Boccio et al. [65] 2021, USA	Retrospective cohort study	BMI	847	None = 69 (57–80) CHF only = 80 (70–87) ESRD only = 61 (51–69) OB only = 63 (54–72) CHF and ESRD = 67.5 (60–73) CHF and OB = 70 (63–80) ESRD and OB = 56 (49–70) CHF, ESRD, and OB = 64 (58–74)	None = 50 CHF only = 56.78 ESRD only = 52.94 OB only = 41.56 CHF and ESRD = 55.88 CHF and OB = 49.57 ESRD and OB = 40 CHF, ESRD, and OB = 40	Compliance with the 30 mL/kg fluid bolus was associated with lower mortality and decreased likelihood of intubation within 72 h of emergency department arrival.
Chen et al. [66] 2022, USA	Retrospective cohort study	BMI	14 467	UW = 66 (56–81) NW = 67 (58–81) OW = 68 (58–79) OB = 64 (55–74)	UW = 49.3 NW = 54.7 OW = 54.7 OB = 46.7	Compared to patients without obesity, those with obesity had lower ICU and hospital mortality rates.
Cinar et al. [35] 2022, Türkiye	Retrospective cohort study	BMI	410	NW = 69.75 ± 16.15 OW = 70.14 ± 15.79 OB *n* = 74.2 ± 11.54 SO = 66.8 ± 9.75	NW = 65 OW = 61 OB = 30 SO = 15	Mortality was lower among patients with a BMI > 25 compared to those with a BMI ≤ 25.
Elkan et al. [67] 2023, Israel	Retrospective cohort study	BMI	1276	Group 1 = 52 (32–67) Group 2 = 64 (49–71) Group 3 = 85 (82–89) Group 4 = 85 (83–88)	Group 1 = 45.3 Group 2 = 50.3 Group 3 = 49 Group 4 = 41.3	BMI more than 25 kg/m^2^ was protective factor for in‐hospital and 90‐day mortality
Lebovitz et al. [29] 2023, Israel	Retrospective cohort study	BMI	90 760	NR	UW = 47.6 NW = 55.1 OW = 46.4 OB class I = 44.9 OB class II = 41.5 OB class III = 35.5	Excluding patients with underweight, higher BMI categories were associated with lower odds of mortality and shorter length of stay compared to those with a BMI in the healthy range weight. This study observed a reverse J‐shaped relationship between BMI and mortality.
Lee et al. [68] 2023, USA	Retrospective cohort study	BMI	9176	No ascites: UW = 50.5 ± 15 NW = 57.4 ± 13.1 OW = 58.7 ± 10.8 OB = 57.5 ± 8.81 Mild ascites: UW = 52.9 ± 15.2 NW = 56.6 ± 11.9 OW = 59.1 ± 8.97 OB = 57.7 ± 8.7 Moderate ascites: UW = 53.4 ± 13.8 NW = 58.8 ± 9.84 OW = 59.3 ± 9.16 OB = 57.4 ± 8.33	No ascites: UW = 50 NW = 52.2 OW = 52.4 OB = 55.6 Mild ascites: UW = 50 NW = 45.9 OW = 55.1 OB = 55.9 Moderate ascites: UW = 44 NW = 54.6 OW = 59.7 OB = 53.5	Among patients with mild and moderate ascites, those with obesity had a reduced hazard of death due to sepsis.
Xu et al. [69] 2023, China	Meta‐analysis	BMI	13 studies	N/A	N/A	Obesity was negatively correlated with in‐hospital, ICU, 30‐day, 90‐day, and 1‐year mortality.
Yeo et al. [6] 2023, South Korea	Prospective cohort study	BMI	6424	NO = 70 (60–79) OB = 70 (60–78)	NO = 54.4 OB = 52.4	Obesity was associated with a decreased risk of in‐hospital mortality.
Studies refuting the obesity paradox						
Arabi et al. [34] 2013, Saudi Arabia	Retrospective cohort study	BMI	2882	UW = 59.1 ± 19.2 NW = 62.2 ± 16.8 OW = 63.5 ± 15.9 OB = 62.2 ± 14.6 SO = 58.4 ± 13	UW = 58.2 NW = 61.8 OW = 61.3 OB = 50.9 SO = 40	Crude hospital mortality was lower in patients with obesity and severe obesity than in those with a BMI in the healthy range, but these associations were not statistically significant after adjusting for baseline characteristics and sepsis interventions.
Rae et al. [45] 2013, USA	Retrospective analysis of multicentre trial	BMI	296	NW = 41.1 ± 15.6 OW = 42.8 ± 17.2 OB = 39.1 ± 14.2 SO = 39.2 ± 12.1	NW = 82 OW = 76 OB = 74 SO = 40	
Wang et al. [26] 2013, USA	Retrospective cohort study	BMI and WC	BMI = 29 966 WC = 30 183	NR	BMI: UW = 30.2 NW = 44.1 OW = 53 OB = 41 SO = 22.3 WC: WC in the healthy range = 55 Large WC = 34.3	Obesity and large WC were associated with an increased risk of sepsis.
Gaulton et al. [49] 2014, USA	Retrospective cohort study	BMI	1779	NO = 60.9 (49.2–71.9) OB = 51.8–69	NO = 63.8 OB = 50.6	BMI was not significantly associated with mortality.
Mica et al. [70] 2014, Switzerland	Retrospective cohort study	BMI	651	NW = 42.9 ± 18.4 OW = 43.4 ± 18.9 OB = 44.3 ± 16.3	NW = 69.8 OW = 85.3 OB = 82	No significant differences in clinical outcomes among the BMI groups, including hospital stay, ICU stay, ventilator days, and mortality.
Gaulton et al. [36] 2015, USA	Retrospective cohort study	BMI	1191	UW = 57.5 (43–69) NW = 57 (44–70) OW = 58 (47–70) OB = 56 (47–66) SO = 50.5 (42–63)	UW = 52 NW = 56.5 OW = 62.8 OB = 48.9 SO = 34.2	Mortality risk did not differ significantly between patients with obesity (including severe obesity) and those with a BMI in the healthy range.
Goode et al. [71] 2016, USA	Correlational study using secondary electronic data	BMI	1917	NR	NR	Higher BMI was independently associated with an increased risk of sepsis after adjusting for length of stay.
Palakshappa et al. [72] 2016, USA	Prospective cohort study	BMI	163	NR	NR	Adiponectin concentrations were lower among patients with obesity and showed an inverse correlation with BMI. However, in the complete adjusted model, adiponectin concentrations were not significantly associated with mortality.
Papadimitriou Olivgeris et al. [42] 2016, Greece	Retrospective cohort study	BMI	834	NO = 56.3 ± 19.8 OB = 59.1 ± 14.8	NO = 67.8 OB = 56.4	Patients with obesity had longer ICU stays and higher ICU mortality compared to those without obesity.
Parker et al. [73] 2016, USA	Retrospective cohort study	BMI	3187	NR	NR	Obesity was independently associated with an increased risk of infection and sepsis/septic shock.
Wong et al. [74] 2017, USA	Retrospective cohort study	BMI	33	NW = 60.1 (29–79) SO = 50.5 (29–65)	NW = 50 SO = 53.8	Hospital and ICU lengths of stay, and mortality rates were similar between patients with and without obesity.
Gameiro et al. [75] 2018, Portugal	Retrospective cohort study	BMI	456	NO = 63.9 ± 16.5 OB = 64.4 ± 14.8	NO = 61.3 OB = 48.8	Obesity was not associated with mortality.
Ji et al. [21] 2018, China	Retrospective cohort study	Body composition parameters	236	No SR or VO = 58 (44–69) VO = 66 (53–74) SR = 76 (65–81) SRO = 75 (69–83)	No SR or VO = 57.8 VO = 72.4 SR = 48.4 SRO = 57.7	Sarcopenic obesity was the only body composition parameter independently associated with an increased risk of 30‐day mortality.
Katayama et al. [76] 2018, Japan	Retrospective cohort study	BMI	569	ABW: UW = 60 (53–69) NW = 69 (62–78) OW = 73 (64–79) OB = 60 (44–72) IBW: UW = 66 (53–77) NW = 69 (61–78) OW = 75 (66–80) OB = 59 (46–70)	ABW: UW = 58.4 NW = 61.5 OW = 50 OB = 61.1 IBW: UW = 63.1) NW = 65 OW = 57.1 OB = 69.2	While no significant mortality differences were observed among BMI subgroups in the IBW group, patients with underweight had higher mortality in the ABW group.
Koch et al. [77] 2018, Germany	Prospective cohort study	BMI	229	NR	NR	Visfatin serum concentrations were associated with sepsis and illness severity, but not with obesity. Elevated levels at ICU admission predicted all‐cause mortality over a two‐year follow‐up.
Kok et al. [38] 2018, Canada	Retrospective cohort study	BMI	362	UW = 53 ± 12.6 NW = 57.7 ± 13.1 OW = 54 ± 13.1 OB = 57.3 ± 9.8 SO = 58.3 ± 10.1	UW = 28.6 NW = 29.5 OW = 46.7 OB = 41 SO = 41.2	Patients with obesity and severe obesity had longer hospital stays and higher in‐hospital mortality than those with a BMI in the healthy range.
Lee et al. [22] 2018, Canada	Retrospective cohort study	VAT/SAT ratio	75	Low VAT/SAT = 38–70 High VAT/SAT = 56–68	Low VAT/SAT = 50 High VAT/SAT = 94.6	Patients with a low VAT/SAT ratio demonstrated higher 90‐day survival rates than patients with a high ratio.
Taylor et al. [78] 2018, USA	Retrospective cohort study	BMI	4126	UW = 65.5 ± 17.6 NW = 64.9 ± 18.8 OW = 64.5 ± 16.5 OB = 61.6 ± 15.5 SO = 57 ± 12.8	UW = 49.2 NW = 45.3 OW = 47.9 OB = 56.9 SO = 69.8	BMI categories was not an independent predictor of mortality.
Tsolakoglou et al. [79] 2020, Greece	Prospective observational study	BMI	744	NR	NR	Higher BMI was significantly associated with an increased risk of central line–associated bloodstream infections.
Weber et al. [33] 2020, USA	Retrospective cohort study	BMI	2019	NO = 62.6 ± 13.6 SO = 57.2 ± 14.1	NO = 49.8 SO = 45.5	No significant differences in length of hospital stay or incidence of postoperative sepsis between patients with severe obesity and those without obesity.
Winter‐Jensen et al. [80] 2020, Denmark	Mendelian randomisation study	BMI	101 447	UW = 57 ± 15 NW = 56 ± 13 OW = 59 ± 13 OB = 60 ± 12	UW = 12 NW = 35 OW = 57 OB = 50	Compared to individuals with a BMI in the healthy range, those with obesity had increased risk of sepsis.
Abumayyaleh et al. [81] 2021, Germany	Retrospective cohort study	BMI	3635	BMI < 25 kg/m^2^ = 59 (18–99) OW = 64 (21–99) OB = 66 (19–98)	BMI < 25 kg/m^2^ = 24.9 OW = 46.1 OB = 29	Patients with obesity had higher mortality compared to those with BMI < 25 kg/m^2^.
Butler‐Laporte et al. [82] 2021, UK	Mendelian randomisation study	BMI	698 BMI‐related single‐nucleotide polymorphisms	NR	NR	Higher BMI was associated with increased 28‐day sepsis mortality and pneumonia mortality.
Ozben et al. [83] 2021, Türkiye	Retrospective cohort study	BMI	147	NO = 63.4 ± 12.3 OB = 66.4 ± 10.5	NO = 61.9 OB = 59.5	No significant difference in postoperative outcomes between BMI groups, including sepsis, hospital stays, and mortality.
Page‐Wilson et al. [41] 2021, USA	Retrospective cohort study	BMI	1019	UW = 75 (48–83) NW = 71 (59–81.5) OW = 65 (54–75.5) OB = 60 (50–71) SO = 52 (40–65.3)	UW = 56 NW = 56.4 OW = 69.7 OB = 50.9 SO = 51.2	Increasing BMI was independently associated with a higher risk of death.
Lameka et al. [40] 2022, USA	Case–control study	BMI	428	NW = 38 OW = 43 OB = 44 SO = 37	NW = 72.4 OW = 75.19 OB = 69.17 SO = 41.5	No significant differences were observed in systemic complications, all‐cause complications, length of stay, or mortality across BMI categories.
Lee et al. [23] 2022, Taiwan	Retrospective database study	ICD‐9‐CM codes	23 898	NO = 67.6 ± 13.26 OB = 64.43 ± 11.7 SO = 62.34 ± 11.26	NO = 49.03 OB = 48.18 SO = 45.43	Severe obesity was independently associated with increased risk of in‐hospital mortality.
Lenney et al. [47] 2022, USA	Retrospective chart review study	BMI	219	BMI < 25 kg/m^2^ = 63 ± 17.1 OW = 63 ± 14.8 OB = 66 ± 13.8 SO = 61 ± 11.7	BMI < 25 kg/m^2^ = 60.1 OW = 57.4 OB = 59.3 SO = 45.5	No significant differences in hospital and ICU length of stay or mortality rates across BMI categories.
Tay‐Lasso et al. [30] 2022, USA	Retrospective cohort study	BMI	1246	NO = 60 (64–85) OB = 54 (60–80)	NO = 68.6 OB = 73.8	Patients with obesity had a similar risk of mortality as those with a BMI in the healthy range.
Tolley et al. [31] 2022, USA	Retrospective cohort study	BMI	7507	NR	NR	Class III obesity was associated with longer ICU stay increased mortality.
Ward et al. [84] 2022, USA	Retrospective cohort study	BMI	1032	UW = 65.1 ± 18.1 NW–OW = 64.8 ± 17.5 OB = 59.6 ± 14.9	UW = 54.3 NW—OW = 52.8 OB = 40.5	Patients with obesity who received less than 30‐by‐3 dosing were associated with longer ICU stays, but not with increased mortality.
Yildiz et al. [37] 2022, Türkiye	Retrospective cohort study	BMI	128	NW = 55.3 ± 0.6 OB = 57.5 ± 11 SO = 58.7 ± 7.2	NR	Compared to patients with a BMI in the healthy range, those with obesity and severe obesity had longer hospital stays and an increased risk of postoperative complication.
Colbran et al. [27] 2023, Australia	Retrospective cohort study	BMI	207	UW = 45.3 ± 0.5 NW = 47 ± 21.5 OW = 51 ± 17.4 OB = 46.3 ± 15.5	UW = 43 NW = 69 OW = 87 OB = 86	No significant differences in mortality and sepsis incidence across BMI categories.
Ning et al. [85] 2023, Germany	Prospective cohort study	BMI	235	Patients without diabetes: NO = 63 (53–76) OB = 61 (54–71) Patients with diabetes: NO = 73 (63–78) OB = (54–74)	Patients without diabetes: NO = 68.2 OB = 70.2 Patients with diabetes: NO = 83.3 OB = 53.3	The progression from non‐sepsis to sepsis and septic shock was associated with significant changes in circulating CD14‐positive monocyte levels, independent of diabetes and obesity.
Nooijer et al. [86] 2023, Greece	Secondary analysis of prospective cohort study	BMI	167	NW = 82 (70–88) OW = 75 (62–81) OB = 67 (61–78)	NW = 52 OW = 29 OB = 45	28‐day mortality was similar between patients across BMI categories. There was a positive correlation between leptin and BMI.
Systematic review studies reported mixed findings on the obesity paradox						
Trivedi et al. [44] 2015, USA	Systematic review	BMI	7 studies	N/A	N/A	Three studies observed significantly decreased mortality in patients with overweight and obesity than those with BMI in the healthy range. Three studies found insignificant association between obesity and mortality. One study found a significant positive association between obesity and mortality in patients with bacteraemia.
Robinson et al. [25] 2020, USA	Robinson et al. [25] 2020, USA	BMI and ICD‐9 codes	9 studies	N/A	N/A	Three studies found that obesity or severe obesity was associated with reduced sepsis‐related mortality. Five studies reported either inconclusive or conflicting evidence regarding the association between obesity and reduced short‐term sepsis mortality.

Abbreviations: ABW, actual body weight; BMI, body mass index; CHF, congestive health failure; ESRD, end‐stage renal disease N/A, not applicable; IBW, ideal body weight; ICD‐9‐CM, the International Classification of Diseases Clinical Modification, 9th Revision; NO, patients without obesity; NR, not reported; NW, patients with a BMI in the healthy range; OB, patients with obesity; OW, patients with overweight; SO, patients with severe obesity; SR, patients with sarcopenia; SRO, patients with sarcopenic obesity; UW, patients with underweight; VAT/SAT, visceral adipose tissue/subcutaneous adipose tissue; VO, patients with visceral obesity; WC, waist circumference.

#### 3.4.2 Measurement Used

Of the 21 studies identifying an obesity paradox, 19 defined obesity using BMI‐based classifications, applying either prespecified BMI or criteria established by the WHO, NIH, or CDC. Two studies used a validated ICD‐9 codes not derived from measured or self‐reported height and weight. [19, 24] In the systematic review by Robinson et al. [25] better survival outcomes for patients with obesity were reported in four studies. Among these, one study used BMI classifications to define obesity, while the other three relied on binary yes/no categorisations based on ICD‐9 coding [19, 24, 48, 50]. In addition, a systematic review by Trivedi et al. [44] observed the obesity paradox in three studies, all of which used BMI categories to define obesity [48, 50, 87].four studies. Among these, one study used BMI classifications to define obesity, while the other three relied on binary yes/no categorisations based on ICD‐9 coding [19, 24, 48, 50]. In addition, a systematic review by Trivedi et al. [44] observed the obesity paradox in three studies, all of which used BMI categories to define obesity [48, 50, 87].

Similarly, 35 research studies and those included in the two systematic reviews that refuted the obesity paradox defined obesity based on BMI measurements. However, this grouping showed more variation in obesity definitions. Four studies employed different metrics, involving ICD‐9 codes for obesity [23], body composition parameters [21], the visceral adipose tissue/subcutaneous adipose tissue (VAT/SAT) ratio [22], and a combination of BMI and waist circumference (WC) [26].

#### 3.4.3 Sample Size

The sample sizes in studies supporting the obesity paradox ranged considerably. Details of study methodologies and findings are shown in Table S1. Some studies demonstrated balanced group sizes. For instance, Lin et al. [63] found a correlation between obesity and lower ICU and 28‐day mortality rates, involving 2664 (33.4%) patients with obesity and 2513 (31%) patients with a BMI in the healthy range. Wacharasint et al. [50] reported reduced 28‐day mortality among 276 patients with BMI < 25 kg/m^2^, 209 with overweight, and 245 with obesity. In addition, Yeo et al. [6] performed propensity score matching to adjust for confounding factors and create equal groups of patients with and without obesity.

Conversely, several studies showed sample size imbalances. Nguyen et al. [24] included 1 551 000 (88%) patients without obesity and only 212 000 (12%) patients with obesity. Similarly, Zhou et al. [60] studied 178 patients, of whom only 11 (6.2%) had obesity, and concluded that survival improved as BMI increased. Sakr et al. enrolled 645 patients (16.5%) with obesity and 113 (2.9%) with severe obesity compared to 1281 (32.8%) patients with a BMI in the healthy range [59]. In contrast, Lee et al. included more participants with obesity than with a healthy BMI when examining the protective effects of obesity in ascites [68]. In this study, patients with a BMI in the healthy range accounted for 13.6%–15% of the sample, while those with obesity represented 55.1%–55.9%.

Inequalities in sample sizes were also evident in studies refuting the obesity paradox. Papadimitriou‐Olivgeris et al. [42] reported that 671 (80.5%) of participants were without obesity, while only 163 (19.5%) were classified as patients with obesity. Lee et al. [23] analysed 21 069 (88.2%) patients without obesity, 1812 (7.2%) with obesity, and 1017 (4.2%) with severe obesity. In a study observed no significant differences in clinical outcomes, Mica et al. [70] included 378 (58.1%) patients without obesity, 224 (34.4%) patients with overweight, and only 49 (7.5%) patients with obesity. Furthermore, several other studies also reported over 70% of participants without obesity compared to 20%–30% with obesity [33, 58, 75, 77, 83].

#### 3.4.4 The Study Population Affected by Obesity

Not all studies reported patient characteristics stratified by BMI categories. Among those that did, most found that 60%–95% of patients were White, except Page‐Wilson et al. [41] who reported only 23.9% (See Table S1). In studies supporting the obesity paradox, eight reported that patients with obesity were younger, more likely to be female, and had higher rates of chronic conditions such as diabetes, hypertension, heart disease, chronic obstructive pulmonary disease, and renal failure than those without obesity or with a BMI in the healthy range [24, 28, 29, 39, 48, 62, 63]. Chen et al. also observed that patients with obesity were younger and more likely to be female but did not report chronic disease distribution [66]. Additionally, four studies found higher male to female ratios and more chronic conditions in patients with obesity but did not observe significant age differences across BMI categories [35, 39, 50, 60].

Alsio et al. [46] observed that patients with obesity were younger and had more chronic conditions, whilst the proportion of females was similar across BMI groups. On the contrary, Elkan et al. [67] reported that among patients aged < 80 years, those with a BMI ≥ 25 kg/m^2^ were older than those with a BMI < 25 kg/m^2^. Both studies found that malignancy rates were higher among patients without obesity compared to those with overweight or obesity [46, 67]. Nguyen et al. [24] also observed that participants without obesity had higher rates of cancer and immunodeficiency syndrome. Only the study by Sakr et al. identified that patients with obesity were more likely to be male and had higher rates of heart failure and diabetes when compared to those in the healthy BMI category, although age distribution was similar across BMI groups [59].

In studies refuting the obesity paradox, nine reported that patients with obesity were more likely to be male than those without obesity [22, 27, 30, 49, 70, 75, 78, 80, 81]. Among these, two studies found that patients with obesity were younger [30, 78], three observed that they were older [22, 80, 81], and four reported similar ages between groups [27, 49, 70, 75]. Conversely, 10 studies demonstrated that patients with obesity were more likely to be female [21, 26, 34, 36, 38, 40, 41, 42, 45, 84]. Of these, five studies identified younger ages for patients with obesity [26, 34, 36, 41, 84], two observed older ages [21, 38, 40], and two found similar ages across groups [42, 45].

Some studies reported that patients with obesity were younger than those without obesity, with no significant differences in gender characteristic [23, 76, 86]. However, five studies included patients with comparable age and gender distributions across BMI categories [33, 47, 56, 74, 83]. Yildiz et al. [37] also observed similar age distributions but did not provide gender data.

Overall, obesity or severe obesity was consistently associated with higher rates of chronic conditions in studies refuting the obesity paradox [23, 26, 27, 30, 33, 34, 36, 38, 41, 42, 47, 49, 56, 75, 78, 81]. Additionally, four studies found that malignancy and/or immunosuppressive disorders were lower in patients with obesity compared to those without obesity [34, 36, 56, 78]. The exception was studies by Wong et al. [74] Ozben et al. [83] and Lameka et al. [40] which found similar rates of chronic diseases between the cohorts.

In reporting baseline characteristics, some studies included severity scores such as Sequential Organ Failure Assessment score (SOFA), Acute Physiology and Chronic Health Evaluation (APACHE), or Simplified Acute Physiology Score (SAPS) to assess illness severity and predict mortality. Among studies refuting the obesity paradox, eight reported no significant differences in severity scores between patients with and without obesity [34, 42, 45, 47, 49, 56, 70, 76]. However, five studies observed higher severity scores among patients with obesity [21, 22, 38, 75, 86]. Specifically, Ji et al. [21] reported that patients with sarcopenic obesity had higher APACHE II and SOFA scores than those with sarcopenia, visceral obesity, or neither condition. Kok et al. [38] found higher APACHE II scores in patients with obesity, but lower scores in those with severe obesity compared to patients with a BMI in the healthy range. Similarly, Nooijer et al. [86] identified increased SOFA scores in patients with obesity, while APACHE II scores were comparable across BMI categories.

In studies supporting the obesity paradox, seven found no significant differences in illness severity among BMI groups [6, 28, 39, 50, 59, 60, 62]. Nonetheless, three studies reported different findings. Lin et al. [63] observed higher SOFA scores in patients with obesity compared to those with a BMI in the healthy range. In contrast, Chen et al. [66] found that patients with obesity had the lowest APACHE IV but similar SOFA scores when compared to those with underweight, a BMI in the healthy range, and overweight. Additionally, Cinar et al. [35] reported significantly higher APACHE II scores in patients with a BMI in the healthy range, whereas SOFA scores were similar across different BMI categories.

## 4 Discussion

This scoping review found that BMI was frequently used as the single metric to define obesity in sepsis research. This methodological approach characterised 90.5% of studies supporting and 88.6% of studies refuting the obesity paradox. Retrospective designs constituted the primary methodological approach, representing 81% of studies supporting and 74.3% of those refuting the paradox. Notable demographic disparities were observed, with a disproportionate distribution of patients across BMI categories. Among studies reporting racial demographics, a consistent overrepresentation of White patients was documented. Studies supporting the obesity paradox consistently identified patients with obesity as disproportionately female, younger, and exhibiting a higher comorbidity prevalence compared to those without obesity, with two studies reporting elevated malignancy rates among patients without obesity. Conversely, studies refuting the paradox demonstrated heterogeneous age and sex distribution while consistently documenting higher rates of chronic conditions among patients with obesity, with exceptions of malignancy and immunosuppressive disorders. Both groups of studies reported comparable or increased illness severity scores in patients with obesity, with a single exception documenting significantly elevated APACHE II scores among patients with a BMI in the healthy range, though SOFA scores remained consistent across BMI categories.

Between 2012 and 2023, BMI has remained the most frequently used method to determine obesity. Obesity is defined by excessive adiposity that poses a health risk, with BMI serving as a surrogate marker for body fat [88]. The ease of BMI calculation is one reason the WHO recommends it as a standard measure for assessing obesity in adults for research purposes [89]. The widespread application of BMI in the studies included in this scoping review may be attributed to the predominance of retrospective study designs, which enable BMI calculation using patients' weight and height data readily available in medical records. However, BMI has limitations, as it can misclassify body fat due to its inability to distinguish between fat, muscle, and bone mass [89].

In addition to generalised obesity measured by BMI, the WHO has emphasised the importance of abdominal obesity, as both types are associated with an elevated risk of morbidity and mortality [90]. Waist circumference (WC), waist‐to‐hip ratio (WHR) and waist‐to‐height ratio (WHtR) are among the anthropometric indicators reflecting abdominal obesity. This scoping review included a study by Wang et al. [26] which found that chronic diseases were more prevalent among people with large WC and high BMI, and that large WC and severe obesity were correlated with an increased risk of sepsis. Likewise, Gurunathan et al. [20] proved that WC was superior to WHR or BMI in predicting postoperative sepsis and mortality. A recent longitudinal study by Lu et al. [91] involving 10 521 middle‐aged and older participants further corroborated the superiority of abdominal obesity indices over BMI. The authors concluded that WHtR, WC, waist divided by height 0.5 (WHT.5R), and BMI were all independent predictors of cardiometabolic multimorbidity. However, WHtR, WC, and WHT.5R exhibited stronger predictive power for future cardiometabolic multimorbidity compared to BMI.

Among studies that defined obesity using BMI, we observed variations in BMI subclassification, such as: (1) patients without obesity versus those with obesity or severe obesity; and (2) patients with underweight, a BMI in the healthy range, overweight, and obesity. A few studies combined categories, such as grouping participants with BMI in the healthy range and underweight together as BMI < 25 kg/m^2^, or those with overweight and obesity as BMI ≥ 25 kg/m^2^. Moreover, several studies further subdivided obesity into Class I, II, III or used alternative descriptors such as obesity and severe obesity. Given that variations in BMI subclassifications influenced sample proportions, they may have contributed to differences in study findings.

Existing studies supporting and refuting the obesity paradox demonstrate inconsistent sample size distributions. Several studies present substantial demographic disparities that warrant methodologiccal scrutiny. Nguyen et al. [24], for instance, included only 12% of patients with obesity compared to 88% of those without obesity, concluding that obesity was associated with improved survival among patients with sepsis. This study, which analysed data from over 1000 hospitals across the United States, raises a significant methodological concern: the representation of individuals with obesity (12%) diverges substantially from the contemporaneous national prevalence of adults with obesity, which was 34.9% [92]. This discrepancy suggests potential sampling bias that may compromise the external validity of the findings and limit generalisability of the study results. Zhou et al. [60] presents another illustrative example of this methodological limitation, wherein only 6.2% of the study cohort (11/178 participants) met the classification criteria for obesity. The authors concluded that 90‐day mortality was inversely associated with BMI. Such a conclusion raises significant epistemological concerns, because it could lead to the assumption that higher BMI (e.g., severe obesity) confers even greater survival benefits in sepsis. This extrapolation beyond the observed data range constitutes a problematic interpretive extension not adequately supported by the limited representation of individuals with obesity within the study sample.

To balance sample sizes between patients with and without obesity, Yeo et al. [6] and Weber et al. [33] performed propensity score matching, which also ensured a balance of baseline characteristics. Propensity matching is a statistical method used to address scepticism that differences in outcomes may be attributed to different patients characteristics, often referred to as an “apples‐to‐oranges” comparison [93]. This balancing score method reassures that the researchers are making valid “apples‐to‐apples” comparisons in non‐randomised cohorts. In their study, Weber et al. found that the incidence of postoperative septic shock, which was significantly higher in the group of patients with severe obesity compared to those without obesity before matching, became no longer significant after matching. This suggests that balancing covariates in the observed cohorts can influence study findings.

This review identified distinct patterns in patient characteristics between the two discourses of interest. In studies supporting the obesity paradox, patients with obesity were generally younger, more likely to be female, had more chronic conditions, but similar severity of illness scores compared to those without obesity. On the other hand, studies refuting the obesity paradox presented more heterogeneous patient characteristics in age, sex, and severity scores. It is imperative to underscore these patterns, given the possible association between patient characteristics and health outcomes. To illustrate, studies investigating the influence of sex on clinical outcomes in patients with sepsis have shown that females had a higher survival rate than males, partly by reason of biological sex differences [94, 95, 96]. In addition, a systematic review and meta‐analysis exploring sex as an independent prognostic factor for mortality, involving 80 520 adult participants, 45.25% of whom were women, found low‐certainty evidence suggesting that female sex may be associated with decreased one‐year all‐cause mortality in patients with sepsis [97].

Researchers must exercise methodological vigilance regarding the potential combination of unidentified confounding variables and disproportionate cohort distributions, which may precipitate the statistical phenomenon known as Simpson's paradox. This paradox manifests when apparent associations observed within discrete subgroups undergo directional reversal upon aggregation of these subgroups [98, 99]. A salient demonstration of Simpson's paradox within obesity research emerges from an investigation examining the relationship between obesity and spontaneous preterm birth (sPTB) risk [100]. When analysing sPTBs occurring between 22 and 36 gestational weeks comprehensively, the data demonstrated increasing adjusted relative risks (aRRs) corresponding to elevated BMI. However, upon stratification of sPTBs according to the presence or absence of obesity‐associated comorbidities, the observed association inverted. Specifically, women with obesity exhibited lower aRRs compared to those with a BMI in the healthy range between 28 and 36 gestational weeks. Further disaggregation by specific comorbidity revealed heterogeneous effects of obesity on both the frequency and aRRs of sPTBs, depending on the comorbidities examined. [100] Therefore, within the context of the obesity paradox discourse, rigorous consideration of methodological constraints, uncontrolled variables, and judicious interpretation of statistically significant associations become imperative. In instances where Simpson's paradox may be operational, failure to implement such analytical rigour may engender misleading inferences.

### 4.1 Strengths and Limitations

Strengths of our review comprise a well‐developed and comprehensive search strategy, rigorous analysis of the literature, and an available published protocol to which we adhered. However, this review has certain limitations. The review did not stratify analyses according to sepsis severity gradations (e.g., sepsis versus septic shock) when evaluating health outcomes. This analytical decision was predicated on the review's primary objective to examine predominant obesity measurement methodologies employed in general sepsis populations over the preceding decade. Another limitation is that some papers provided insufficient patient characteristics, which prevented us from presenting complete data across all included studies. This constraint potentially limits the robustness of certain comparative analyses and may attenuate the strength of specific conclusions.

### 4.2 Clinical Implications and Future Research

This review found that BMI is the predominant measure used to define obesity in clinical studies, despite its inability to distinguish fat mass from other components of body mass. As an anthropometric measure, BMI serves both as a screening tool for obesity and, following diagnosis, as a classification system for determining the levels of obesity. However, growing evidence supports the existence of distinct obesity phenotypes, such as metabolically healthy obesity and normal weight obesity, each characterised by different health risks related to body fat and metabolic disorders [101]. Referring to these phenotypes, BMI is not an accurate indicator of obesity, as individuals may have obesity‐related health risks regardless of their BMI and may be healthy across a range of BMI levels. An emerging classification is clinical obesity, which considers not only excess adiposity but also the presence of activities of daily living limitations related to obesity [14]. The reliance on BMI per se to define obesity is problematic, as obesity is not solely a matter of body size. It involves complex metabolic and immunological processes that underpin pathophysiological effects and inform diagnostic classification.

Our concerns with using BMI as a screening tool for obesity arise from limitations in accurately defining obesity in patients with inflammatory conditions such as sepsis, which may contribute to the observed obesity paradox. The lack of research into the methodological rigour with which the definitions of obesity are applied to the patient population within clinical studies may be attributable to the occurrence of the obesity paradox.

The findings of this scoping review underscore the need for both a systematic review and prospective studies to examine how different definitions of obesity, whether based on anthropometric, metabolic, and inflammatory measures, influence clinical outcomes in patients with sepsis. Identifying a more clinically appropriate definition of obesity is particularly important for managing patients with acute inflammatory conditions. Therefore, this study is relevant to researchers and healthcare professionals working in the acute clinical unit, where patients with obesity are at increased risk of deterioration.

## 5 Conclusion

This scoping review identified two conflicting perspectives on the obesity paradox in sepsis: supporting and refuting the obesity paradox. The predominant methodological approach in studies of obesity and health outcomes in sepsis involved using body mass index as the operational definition of obesity and applying retrospective study designs. Key methodological concerns include the reliance on BMI and the comparability of cohort characteristics, both of which substantially affect the interpretation of research outcomes. Prospective studies employing obesity metrics that account for pathophysiological characteristics of adiposity are needed to improve patient care. Moreover, future research should systematically investigate alternative obesity classification schemas and elucidate their differential impacts on clinical outcomes.

## Author Contributions

Study conceptualisation and methodology: E.K., C.H., B.R. Literature search: E.K. Screening: E.K., C.H., B.R. Data extraction: E.K. Checking data extraction: C.H. and B.R. Writing – original draft: E.K. Writing – review and editing: C.H. and B.R. All authors contributed to the revision of the manuscript and approved the final version.

## Conflicts of Interest

The authors declare no conflicts of interest.

## Supporting information


**Data S1:** Supporting Information.

## Data Availability

The data that supports the findings of this study are available in the Supporting Information of this article.
