# Supplementary material for: Do Obesity Classifications Create the Obesity Paradox? A Scoping Review of Obesity Definitions Applied in Sepsis Research
Source: Clin Obes. 2025 Aug 8;16(1):e70039. doi: 10.1111/cob.70039 (PMC12705256; doi:10.1111/cob.70039)
Supplement: Supplementary file 1 — Data S1: Supporting Information. [file COB-16-e70039-s001.pdf]

# Do obesity classifications create the obesity paradox? A scoping review of obesity definitions applied in sepsis research

Efris Kartikasari[1], Brian Robinson[1], Caz Hales[1]

[1]School of Nursing, Midwifery, and Health Practice, Victoria University of Wellington, Wellington, New Zealand

**Corresponding author**:

Efris Kartikasari, School of Nursing, Midwifery, and Health Practice, Victoria University of Wellington, Level 7 Clinical Services Block, Wellington Regional Hospital, 39 Riddiford St, Newtown, Wellington 6021, New Zealand. Email: efris.kartikasari@vuw.ac.nz

**Supporting information**

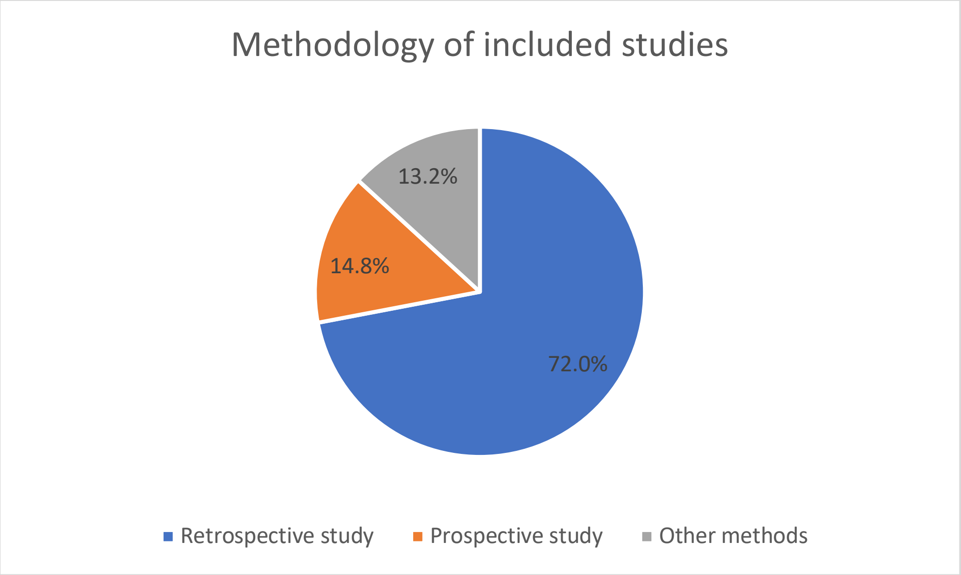


Figure S1 Study designs of reviewed studies.

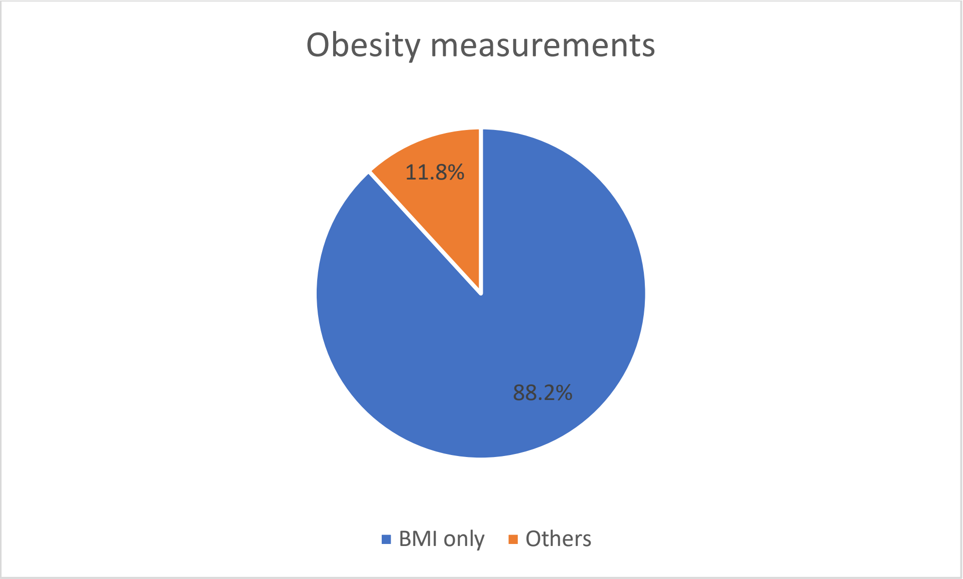


Figure S2. Obesity assessments in included studies.

Table S1. Detailed findings of included studies

| Author, year, country | Methodology | Sample size and groups, n (%) | Findings |
|---|---|---|---|
| | | **Studies supporting the obesity paradox** | |
| Sakr et al., 2012, Italy | Retrospective cohort study<br>Defined obesity according to the NIH and WHO criteria | N = 3,902<br>UW = 102 (2.6%)<br>NW = 1,281 (32.8%)<br>OW = 1,761 (45.1%)<br>OB = 645 (16.5%)<br>SO = 113 (2.9%) | Patients with overweight and obesity were more likely to be male (74.2% and 58.1%, respectively) compared to those with a BMI in the healthy range (55%) ($p < 0.01$).<br>Age, SAPS II score, and incidence of sepsis on admission to the ICU were similar between BMI groups.<br>Heart failure (NYHA class III-IV) was more prevalent in patients with obesity (11.8%) and severe obesity (15%) than those with a BMI in the healthy range (6.4%), and diabetes increased significantly with increasing BMI categories.<br>ICU length of stay was longer in patients with severe obesity (5 (2-12.5) vs. 3 (1-9) days, $p = 0.003$) and shorter in patients with underweight (2 (1-5) vs. 3 (1-9) days, $p = 0.006$) than those with a BMI in the healthy range.<br>Severe obesity was associated with an increased risk of death in ICU patients after elective surgery (OR 4.63, 95% CI 1.07-19.87, $p = 0.039$).<br>Patients with overweight and obesity had a lower risk of ICU death (OR 0.73, 95% CI 0.58-0.91, $p = 0.007$ and OR 0.62, 95% CI 0.45-0.85, $p = 0.003$, respectively).<br>Underweight was associated with increased ICU mortality for both short-term monitoring (OR 3.92, 95% CI 1.34-11.43, $p = 0.012$) and after elective surgery (OR 5.58, 95% CI 1.81-17.22, $p = 0.003$). |
| Kuperman et al., 2013, USA | Retrospective cohort study<br>Defined obesity using the WHO criteria | N = 792<br>UW = 49 (6%)<br>NW = 261 (33%)<br>OW = 249 (31%)<br>OB = 187 (24%)<br>SO = 46 (6%) | 92% of the patients were White.<br>Age was similar between BMI groups.<br>Patients with higher BMI were more likely to have diabetes and COPD ($p < 0.01$ and 0.04, respectively).<br>Patients with underweight or severe obesity were more likely to be female (65.3% and 73.9%, respectively).<br>No significant difference in disease severity, as measured by the APACHE II score, across the groups.<br>Diabetes was protective factor for mortality (OR 0.52, 95% CI, 0.29-0.93, $p = 0.03$).<br>Patients with underweight had increased inpatient mortality (OR 1.5, 95% CI 0.67-6.3).<br>Severe obesity was protective for inpatient sepsis mortality (OR 0.7, 95% CI 0.12-4.2).<br>Survivors had higher BMI (27.6 $kg/m^2$) compared with non-survivors (26.3 $kg/m^2$), $p = 0.03$. |
| Wacharasint et al., 2013, Canada | Retrospective cohort study<br>Defined obesity using the NIH criteria | N = 730<br>BMI < 25 $kg/m^2$ = 276 (37.8%)<br>OW = 209 (28.6%)<br>OB = 245 (33.6%) | Patients with obesity had a lower proportion of male patients (55.5%) than those with overweight (67.9%) and BMI < 25 $kg/m^2$ (62%), $p = 0.03$.<br>The patients' ages were comparable across groups ($p = 0.62$).<br>More than 80% of the patients were Caucasian; no significant racial differences among groups.<br>Patients with obesity had a higher rate of diabetes (29.8%) than patients with overweight (20.6%) and BMI < 25 $kg/m^2$ (14.9%), $p < 0.0001$).<br>Severity of illness (APACHE II score) was comparable among groups. |

| | | | |
|---|---|---|---|
| | | | Patients with obesity had the highest serum creatinine concentration (91 µmol/L) than those with overweight (150 µmol/L) and BMI < 25 kg/m$^2$ (130 µmol/L), p < 0.0001.<br>Patients with overweight and obesity had markedly lower rate of lung (OB 35%, OW 45%, and BMI of < 25 kg/m$^2$ 49.8%; p = 0.003) and fungal infections (OB 8.2%, OW 11%, and BMI < 25 kg/m$^2$ 15.6%; p = 0.03).<br>Compared with patients with a BMI of less than 25 kg/m2, patients with overweight and obesity had a lower 28-day mortality (p = 0.02).<br>For every 1-unit increase in BMI, HR-adjusted mortality decreased by 2% (95% CI 0.97-0.99, p = 0.04). |
| Prescott et al., 2014, USA | Retrospective cohort study<br>Defined obesity using the WHO classification | N = 1,404<br>NW = 597 (42.5%)<br>OW = 473 (33.7%)<br>OB = 202 (14.4%)<br>SO = 132 (9.4%) | Patients with obesity and severe obesity were younger (p < 0.001), predominantly female (p < 0.001), more likely to have diabetes (p < 0.001), and had higher rates of acute renal dysfunction (p = 0.001) relative to three other BMI groups.<br>The majority of patients were White, non-Hispanic (NW 76.6%, OW 68.5%, OB 68.2%, and SO 68.1%).<br>Compared with patients with a BMI in the healthy range, those with obesity (OR 0.53, 95% CI 0.35-0.79) and severe obesity (OR 0.43, 95% CI 0.25-0.74) had lower odds of 90-day mortality.<br>Patients with obesity and severe obesity also had a lower risk of 1-year mortality (OR 0.59, 95% CI 0.39-0.88 and OR 0.46, 95% CI 0.26-0.80, respectively). |
| Abbate et al., 2016, USA | Secondary analysis of study<br>Defined obesity according to the validated ICD-9-based chronic disease indicator | N = 116,566 | Of all visits, 13,991 (12.0%) were coded as visits by patients with obesity.<br>During the hospital admission, 30,712 visits (26.3%) resulted in death.<br>Obesity was inversely associated with in-hospital mortality (OR 0.74, 95% CI 0.71-0.77).<br>Obesity was associated with lower mortality in adults aged 50 and older (OR 0.65, 95% CI 0.62-0.68, p for interaction < 0.001), but not in those under 50 (OR 0.99, 95% CI 0.87-1.13). |
| Nguyen et al., 2016, USA | Retrospective cohort study<br>Defined obesity with ICD-9-CM codes | N = 1,763,000<br>NO = 1,551,000 (88%)<br>OB = 212,000 (12%) | Patients with obesity were younger compared to those without obesity (62 (52-71) years vs. 70 (56-81) years, p < 0.001).<br>Patients with obesity had a higher proportion of female than those without obesity (59.7% and 49.4%, respectively, p < 0.001).<br>Patients with obesity had significantly higher rates of CHF, pulmonary disorders, diabetes, hypertension, chronic pulmonary diseases, liver diseases, renal failure, depression, psychoses, deficiency anaemia, arthritis, and hypothyroidism.<br>Patients without obesity showed markedly higher rates of AIDS, substance abuse, fluid and electrolyte disorders, coagulopathy, cancer, valvular diseases, paralysis, and neurological disorders.<br>Obesity was related to a 16% reduction in mortality among hospitalised patients with sepsis (OR 0.84, 95% CI 0.81-0.88), remaining significant across sepsis subcategories, including sepsis as the principal diagnosis, severe sepsis, and septic shock. |

| | | | |
|---|---|---|---|
| | | | Patients with obesity had a slightly longer hospital stay than those without obesity (adjusted difference = 0.65 days, 95% CI 0.44-0.86), with the trend remaining significant across sepsis subcategories (sepsis, severe sepsis, or septic shock) and discharge outcomes (alive/dead). |
| Zhou et al., 2018, China | Prospective cohort study<br>Defined obesity using the WHO criteria | N = 178<br>UW = 33 (18.5%)<br>NW = 98 (55.1%)<br>OW =36 (20.2%)<br>OB = 11 (6.2%) | Patients with obesity were less likely to be male (p = 0.002) and had higher rates of COPD (p = 0.017) but lower rates of septic shock (p = 0.004), in-hospital mortality (p = 0.027), and 90-day mortality (0.015) compared to those in the other BMI categories.<br>The patients' ages were comparable across BMI groups.<br>No significant differences in SOFA and APACHE II scores across the groups.<br>BMI (HR 0.940, 95% CI 0.889-0.994, p = 0.029) was identified as independent factors for 90-day death.<br>The Kaplan-Meier curve for 90-days survival displayed that higher BMI was associated with improved prognosis (p = 0.008) among patients with sepsis admitted to the ICU. |
| Pepper et al., 2019, USA | Retrospective cohort study<br>Defined obesity using the NIH criteria | N = 55,038<br>UW = 3,302 (6%)<br>NW = 18,163 (33%)<br>OW = 15,411 (28%)<br>OB class I = 8,806 (16%)<br>OB class II = 4,403 (8%)<br>OB class III = 4,953 (9%) | The majority of patients were White (n = 41,671) and aged 65 years or older (n = 33,187).<br>Compared to patients with a within the healthy range, the odds of short-term mortality were higher in those with underweight (OR 1.62, 95% CI 1.50–1.74), and lower in those with overweight (OR 0.73, 95% CI 0.70–0.77), Class I obesity (OR 0.61, 95% CI 0.57–0.66), Class II obesity (OR 0.61, 95% CI 0.55–0.67), and Class III obesity (OR 0.65, 95% CI 0.59–0.71) (p < 0.0001).<br>Significant interactions were found between BMI and sex (p = 0.035), and BMI and albumin quartile (p = 0.001), but not with age, ethnicity, and SOFA quartile.<br>Among patients with albumin levels below 2 g/dL, patients with overweight and Class I obesity had improved survival but not those with Class II or Class III obesity. |
| Jagan et al., 2020, USA | Retrospective cohort study<br>Defined obesity using the CDC criteria | N = 7,967<br>UW = 392 (4.9%)<br>NW = 2,281 (28.6%)<br>OW = 2,160 (27.1%)<br>OB = 3,134 (39.4%) | Compared to patients in without obesity, those with obesity were younger (64 (53–74) years, p < 0.001), more likely to have a MAP ≥ 65 mm Hg (92%, p = 0.029), and had higher proportions of diabetes (49.3%, p < 0.001), history of heart failure (28.7%, p < 0.001), and elevated creatinine levels (1.3 mg/dL (1.0–1.9), p < 0.001).<br>A higher MAP (OR 0.99; 95% CI, 0.98–0.99) and the presence of diabetes (OR 0.71; 95% CI, 0.60–0.84) were protective against inpatient mortality, while older age was not (OR 1.04; 95% CI, 1.04–1.05).<br>No significant differences in APACHE III score among the groups.<br>Unadjusted in-hospital mortality rate was lower for higher BMI categories (OR 12.1%, 95% CI 11.4%-12.8%, p < 0.007). Mortality rates by obesity class were 9.9% for Class 1 (95% CI 8.5%-11.6%; n = 1,390), 9.1% for Class 2 (95% CI 7.3%-11.3%; n = 815), and 5.8% for Class 3 (95% CI 4.4%-7.6%; n = 929).<br>The obesity paradox was evident only in patients with MAP ≥ 65 mm Hg (BMI, p < 0.001 for both lactate ≤ 2 mmol/L and > 2 mmol/L). Including the APACHE III score as covariate (n = 844) showed no significant mortality differences between BMI categories (p = 0.472). |

| | | | |
|---|---|---|---|
| Kotecha et al., 2020, USA | Retrospective cohort study<br>Defined obesity according to BMI | N = 2,017<br>UW = 145 (7.2%)<br>NW = 1,406 (69.7%)<br>SO = 466 (23.1%) | The majority of patients (89.8%) were Caucasian.<br>Patients with a BMI in the healthy range had a higher proportion of male patients (55.8%) than those with underweight (43.5%) and obesity (53.9%), p = 0.02.<br>Patients with severe obesity were younger (61.7 (53.2-69.6) years) than those with a BMI in the healthy range (65.3 (54.1-76.1) years) and underweight (62.4 (50.5-71.7) years).<br>Patients with obesity had significantly higher rates of CHF and diabetes, while patients with underweight more frequently had a history of COPD.<br>No significant differences in SOFA and APACHE III scores among the BMI groups.<br>Total norepinephrine exposure, highest in patients with severe obesity, was an independent predictor of in-hospital and 1-year mortality (OR 2.2, 95% CI 1.8-2.6, p < 0.001).<br>A higher BMI showed a protective effect against both in-hospital mortality (OR 0.2, 95% CI 0.1-0.3, p < 0.001) and 1-year mortality (OR 0.3, 95% CI 0.2-0.6, p < 0.001).<br>In-hospital mortality decreased with higher BMI (UW 41.4%, NW 28.4%, SO 24.7%; p < 0.001), but this trend was not seen for 1-year mortality. Patients with severe obesity had higher 1-year mortality than those with a BMI in the healthy range and underweight. |
| Lin et al., 2020, Israel | Retrospective cohort study<br>Defined obesity using the WHO criteria | N= 7,967<br>UW = 325 (4.1%)<br>NW = 2,513 (31.5%)<br>OW = 2,465 (31%)<br>OB = 2,664 (33.4%) | The mean age of patients overweight (66.51 ± 16.21) and obesity (63.71 ± 14.36) was lower compared to those with a BMI in the healthy range (67.56 ± 16.88) and underweight (67.02 ± 17.6).<br>Patients with obesity had a slightly higher proportion of females (47.52% vs. 45.76%) and a lower proportion of males (52.48% vs. 54.24%) compared to those with a BMI in the healthy range.<br>The median SOFA scores were higher in patients with overweight and obesity (6.00 (4.00-8.00) in both groups), than that of patients with a BMI in the healthy range and underweight (5.00 (3.00-8.00) and 4.00 (3.00-8.00), respectively).<br>Diabetes and CHF were more prevalent among patients with obesity compared to those in other BMI categories (p < 0.001).<br>Patients with obesity had longer ICU and hospital stays (p < 0.001).<br>Among male patients, those with overweight and obesity had a lower risk of 28-day mortality (HR 0.74, 95% CI 0.63-0.86, p = 0.0001 and HR 0.63, 95% CI 0.53-0.76, p < 0.0001, respectively).<br>Twenty-eight-day mortality was 22% lower in patients with obesity (HR 0.78, 95% CI 0.69-0.88, p = 0.0001) and 13% lower in patients with overweight (HR 0.87, 95% CI 0.78-0.98, p = 0.0263) compared to those with a BMI in the healthy range.<br>The risk of ICU mortality was lower in patients with obesity (OR 0.77, 95% CI 0.65-0.91, p = 0.0023) than patients with a BMI in the healthy range. |

| | | | |
|---|---|---|---|
| Shimada et al., 2020, Canada | Retrospective cohort study<br>Defined obesity based on BMI | N = 519<br>25≥ BMI = 178<br>25< BMI = 312 | Twenty-eight-day survival increased with the number of minor rs7852409 C alleles (VLDLR GOF allele), from 0 (GG) to 1 (GC) to 2 (CC) (HR: 0.662, 95% CI 0.477–0.918, p= 0.014).<br>The survival effect of the VLDLR genotype was significant in patients with gram-negative infection (p = 0.003; hazard of death for C allele carriers compared with GG homozygotes: 0.28; 95% CI, 0.12-0.64) and mostly confined to this group (interaction p = 0.017).<br>Patients with obesity have more adipose tissue and therefore a higher total number of adipocytes VLDLR. |
| Alsio et al., 2021, Sweden | Prospective cohort study<br>Defined obesity according to the WHO criteria | N = 1,656<br>UW = 54 (3.3%)<br>NW = 625 (37.7%)<br>OW = 602 (36.4%)<br>OB = 251 (15.2)<br>SO = 124 (7.5%) | BMI inversely correlates with age (p = 0.002).<br>The proportion of female patients was similar across BMI groups.<br>As BMI increased, the proportion of patients with diabetes, cardiovascular disease, and CRF significantly increased, while the proportion of those with malignancies decreased.<br>Age and existing malignancy were independent predictors for 28-day and one-year case fatality rate (CFR), while CRF was a significant predictor for one-year CFR.<br>Higher BMI was associated with lower 28-day (OR 0.93, 95% CI 0.88-0.98, p = 0.009) and one-year case fatality rate (OR 0.94, 95% CI 0.91-0.97, p < 0.001). |
| Boccio et al., 2021, USA | Retrospective cohort study<br>Defined obesity using the WHO criteria | N = 847<br>None, n = 308 (36.4%)<br>CHF only, n = 199 (23.5%)<br>ESRD only, n = 17 (2%)<br>Obesity only, n = 154 (18.2%)<br>CHF and ESRD, n = 34 (4%)<br>CHF and OB, n = 115 (13.5%)<br>ESRD and OB, n = 5 (0.6%)<br>CHF, ESRD, and OB = 15 (1.8%) | The median age of patients was 70 years (59-81), and the majority of them were male (50.6%) and White (74.6%).<br>All groups, except patients with obesity only and patients with ESRD and obesity, were less likely to receive at least 2000 cc of total volume compared to those without pre-existing conditions.<br>Total volume-based goal compliance was lower in patients with multiple pre-existing conditions (OR 0.08, 95% CI 0.03–0.26; p < 0.001) and decreased as the number of conditions increased.<br>Provider compliance with the 30-cc/kg bolus was associated with decreased death among patients with obesity (OR 0.47, 95% CI 0.25-0.90, p = 0.02) and no pre-existing condition (OR 0.44, 95% CI 0.27-0.74, p < 0.01).<br>Among patients with septic shock and coexisting CHF - specifically those with CHF, ESRD, obesity, and no pre-existing conditions - provider compliance was associated with a reduced mean ICU LOS. |
| Chen et al., 2022, USA | Retrospective cohort study<br>Defined obesity using the WHO classification | N = 14,467<br>UW = 827 (5.7%)<br>NW = 4,626 (32%)<br>OW = 3,808 (26.3%)<br>OB = 5,206 (36%) | The racial distribution of patients was Caucasian (78.4%), American (11.2%), and other (10.3%).<br>Patients with obesity were younger (64 (55.0-14.0) years, p < 0.001), and had a lower proportion of male (46.7%, p < 0.001) than those in the other BMI groups.<br>Patients with obesity had the lowest APACHE IV score (< 0.001) but a similar SOFA score (p = 0.368) when compared to the other groups. |

| | | | |
|---|---|---|---|
| | | | Patients with obesity had lower ICU mortality (8.8%, p < 0.001) and hospital mortality (13.9%, p < 0.001) rates.<br>Patients with obesity had longer median ICU LOS, at 2.97 (1.87-5.56) days, p < 0.001<br>Patients with obesity had higher ICU prolonged LOS (ICU p-LOS) (p < 0.001).<br>Patients with prolonged ICU LOS were more likely to have COPD, CKD, liver disease, hypertension, and CHF than those with non-prolonged.<br>Prolonged ICU LOS was an independent risk factor for ICU mortality (OR = 1.41, 95% CI 1.04–1.91, p = 0.026). |
| Cinar et al.**,** 2022, Türkiye | Retrospective cohort study<br>Defined obesity using the WHO classification | N = 410<br>NW = 170 (42%)<br>OW = 132 (32%)<br>OB = 83 (20%)<br>SO = 25 (6%) | Patients with severe obesity and obesity had a higher proportion of females (85% and 70%, respectively) than those with overweight (32%) and a BMI in the healthy range (42%) (p < 0.001).<br>The patients' ages were comparable across BMI categories (p = 0.068).<br>Patients with a BMI in the healthy range had significantly a higher APACHE II score, pneumonia, and malignancy than those in the other groups. There were no significant differences in SOFA scores across the BMI groups.<br>Patients with obesity and severe obesity had higher rates of diabetes, CAD, hypertension, respiratory failure, and asthma exacerbation compared to those without obesity.<br>Non-invasive ventilation use was higher in patients with obesity and severe obesity (p = 0.017).<br>Sepsis was identified in 113 (28%) cases: 32% of patients with a BMI in the healthy range, 26% of those with overweight, 24% with obesity and 20% with severe obesity (p = 0.427). Ninety-three patients (23%) died of them: 27% were patients with a BMI in the healthy range, 24% with overweight, 15% with obesity, and 12% with severe obesity (p = 0.05). |
| Elkan et al.**,** 2023, Israel | Retrospective cohort study<br>Defined obesity using the WHO criteria | N= 1,276<br>Group 1 = age < 80 years and BMI < 25 kg/m2 = 413 (32.4%)<br>Group 2 = age < 80 years and BMI ≥ 25 kg/m2 = 517 (40.5%)<br>Group 3 = age ≥ 80 years and BMI < 25 kg/m2 = 145 (11.4%)<br>Group 4 = age ≥ 80 years and BMI ≥ 25 kg/m2 = 201 (15.7%) | Patients in group two were significantly older and had more comorbidities than patients in group one. Patients in group four had more diabetes and less malignancy than those in group three.<br>No significant differences in the proportion of females between the groups.<br>Age > 80 was a risk factor for 30- and 90- day mortality (OR 1.73, 95% CI 1.01-2.96, p= 0.44 and OR 1.96, 95% CI 1.24-3.10, p= 0.004).<br>BMI more than 25 kg/m$^2$ was protective factor for in-hospital and 90-day mortality (OR 0.51, 95% CI 0.30-0.86, p= 0.012, and OR 0.57, 95% CI 0.36-0.89, p= 0.015).<br>No significant interaction between age > 80 and BMI > 25 kg/m$^2$ in all mortality outcomes suggests the protective effect of overweight and obesity apply to all age groups. |

| | | | |
|---|---|---|---|
| Lebovitz et al., 2023, Israel | Retrospective cohort study<br>Defined obesity based on BMI | N = 90,760<br>UW = 14,440 (15.9%)<br>NW = 11,170 (12.3%)<br>OW = 9,260 (10.2%)<br>OB class I = 12,595 (13.9%)<br>OB class II = 9,450 (10.4%)<br>OB class III = 33,845 (37.3%) | Patients with obesity were more likely to be female (p < 0.001) and younger than those without obesity (p < 0.001).<br>Compared to patients without obesity, those with obesity (Class I, II, and III) had significantly higher comorbidity rates including COPD, diabetes, CRF, atrial fibrillation/flutter (p < 0.001)<br>Higher Deyo-CCI scores were observed in patients with Class III obesity.<br>Male sex, older age, increasing Deyo-CCI score, CRF, atrial fibrillation/flutter, and CHF were predictors of in-hospital mortality (p < 0.001) and were associated with longer LOS (p < 0.001).<br>Higher mortality rate was observed in patients with underweight (13.7%) and a BMI in the healthy range (10.7%) (p < 0.001). Longer LOS was documented in patients with underweight (10.74 ± 0.21 days), a BMI in the healthy range (9.99 ± 0.19 days), and overweight (9.08 ± 0.19 days) (p < 0.001).<br>Patients with underweight had the highest mortality risk (OR 1.35, 95% CI 1.25-1.47, p < 0.001).<br>Patients with underweight had a longer mean LOS than those with Class III obesity (9.73, 95% CI 9.34-10.12, p < 0.001 vs. mean 7.34, 95% CI 7.05-7.63, p < 0.001, respectively).<br>Excluding underweight, all other BMI ranges were associated with lower odds of mortality (p < 0.001) and shorter LOS (p < 0.001). |
| Lee et al., 2023, USA | Retrospective cohort study<br>Defined obesity using the WHO criteria | No ascites, n = 1,188<br>UW = 4 (0.3%)<br>NW = 161 (13.6%)<br>OW = 359 (30.2%)<br>OB = 664 (55.9%)<br>Mild ascites, n = 4,463<br>UW = 28 (0.6%)<br>NW = 643 (14.4%)<br>OW = 1,311 (29.4%)<br>OB = 2,481 (55.6%)<br>Moderate ascites, n = 3,525<br>UW = 25 (0.7%)<br>NW = 529 (15%)<br>OW = 1,030 (29.2%)<br>OB = 1,941 (55.1%) | Among patients without ascites, there were no significant differences in the proportion of male patients. Patients with obesity were slightly older (57.50 ± 8.81 years) compared to those with a BMI in the healthy range (57.40 ± 13.10) (p = 0.05). The racial profile also differed significantly, with 81.9% of patients with obesity identifying as White compared to 70.8% of those with a BMI in the healthy range (p = 0.01). Additionally, patients with obesity had a higher prevalence of diabetes (50.9% vs. 31.7%, p < 0.001). Despite these differences, Patients with obesity had a lower MELD score (20.20 ± 8.73) compared to those with a BMI in the healthy range (21.80 ± 8.97, p < 0.001).<br>Among patients with mild ascites, no significant differences in age (p = 0.61) or MELD scores (p = 0.83) between those with obesity and a BMI in the healthy range. However, patients with obesity had a higher proportion of male recipients than the reference group (55.90 vs 45.90%, p < 0.001). The racial profile also differed significantly, with 83.8% identifying as White among patients with obesity compared to 73.7% of those with a BMI in the healthy range (p < 0.001). In addition, compared with patients with a BMI in the healthy range, those with obesity had a higher proportion of recipients with diabetes (51.40 vs 31.60%, p < 0.001).<br>Among patients with moderate ascites, patients with obesity were younger (57.40 ± 8.33 years) compared to those with a BMI in the healthy range (58.80 ± 9.84, p < 0.001). The racial distribution also varied significantly, with 80.9% of patients with obesity identified as White compared to 76.2% of those with a BMI in the healthy range (p < 0.001). However, the proportion of patients with diabetes was higher among patients with obesity (52.2%) compared to those with a BMI in the healthy range (37.8%, p < 0.001). Additionally, MELD scores were greater in patients with obesity (26.20 ± 8.71) than in those with a BMI in the healthy range (25.20 ± 8.56, p = 0.03). |

| | | | |
|---|---|---|---|
| | | | In the final model, patients with obesity showed a significantly lower rate of all-cause mortality during the 200 -2012 period (HR 0.77, 95% CI 0.60-1.00, p = 0.05). Moreover, among those with moderate ascites, patients with obesity showed a reduced hazard of death due to general infectious causes (HR 0.44, 95% CI 0.26–0.74, p = 0.002) and sepsis (HR 0.54, 95% CI 0.29-1.00, p = 0.05).<br>From 2012-2019, Patients with obesity and mild ascites experienced a reduced risk of all-cause mortality (HR 0.71, 95% CI 0.52-0.97, p = 0.03). Among patients with mild ascites, patients with obesity also had a reduced hazard of death from general infectious causes (HR 0.42, 95% CI 0.22-0.80, p = 0.009) and sepsis (HR 0.37, 95% CI 0.17-0.82, p = 0.01). |
| Xu et al., 2023, China | Meta-analysis<br>Defined obesity based on BMI | N = 13 studies (11 retrospective cohort studies and 2 prospective cohort studies) | Overweight was associated with reduced ICU mortality (OR [95% CI] = 0.89 [0.80, 0.98], p = 0.014), 30-day mortality (0.84 [0.72, 0.98], p = 0.029), 90-day mortality (0.70 [0.62, 0.79], p = 0.029), and 1-year mortality (0.80 [0.71, 0.90], p < 0.001).<br>Obesity was negatively correlated with in-hospital (OR 0.85, 95% CI 0.75-0.97, p = 0.016), ICU (OR 0.84, 95% CI 0.76-0.93, p = 0.001), 30-day (OR 0.65, 95% CI 0.56-0.76, p = < 0.001), 90-day (OR 0.56, 95% CI 0.46-0.67, p = < 0.001), and 1-year (OR 0.67, 95% CI 0.60-0.76, p < 0.001) mortality.<br>Underweight was associated with increased in-hospital (OR 1.24, 95% CI 1.08, 1.43, p = 0.003), ICU (OR 1.16, 95% CI 0.71, 1.90, p = 0.559), and 1-year (OR 1.55, 95% CI 1.21, 1.99, p = < 0.001) mortality. |
| Yeo et al., 2023, South Korea | Prospective cohort study<br>Defined obesity using the WHO criteria | N = 6,424<br>Before propensity matching: OB (1,335 (20.8%)) vs. NO (5,089 (79.2%))<br>After matching (1:1 ratio): OB (1,335 (50%)) vs. NO (1,335 (50%)) | There were no significant differences in baseline characteristics (e.g. age, male sex, comorbidities, SOFA score, septic shock) between patients with and without obesity after propensity score matching.<br>Patients with obesity had significantly lower In-hospital mortality than those without obesity; obesity was associated with a decreased risk of in-hospital mortality (HR 0.78, 95% CI, 0.68-0.90, p = 0.001).<br>Relative to patients with a BMI in the healthy range, the adjusted OR for hospital mortality was 1.25 (p = 0.004) for patients with underweight, 0.58 (p < 0.001) for patients with overweight, and 0.70 (p = 0.047) for patients with obesity.<br>Among patients admitted to the ICU, hospital mortality was lower among patients with obesity (HR 0.83 95% CI 0.70-0.98, p = 0.031) compared to those without obesity. |
| **Studies refuting the obesity paradox** | | | |
| Arabi et al., 2013, Saudi Arabia | Retrospective cohort study<br>Defined obesity using the WHO classification | N = 2,882<br>UW = 196 (6.8%)<br>NW = 1,020 (35.3%)<br>OW = 816 (28.3%)<br>OB = 680 (23.6%)<br>SO = 170 (5.4%) | Patients with severe obesity and underweight were younger than those with a BMI in the healthy range (58.4 ± 13.0 and 59.1 ± 19.2 years vs. 62.2 ± 16.8 years, p < 0.001).<br>Patients with obesity and severe obesity were more likely to be female (p < 0.001), have heart failure (p = 0.002) and diabetes mellitus (p < 0.001, and less likely to have immunosuppressive disorders (p = 0.06) compared to those with a BMI in the healthy range.<br>No significant differences in APACHE II score across the BMI groups.<br>The crude hospital mortality of patients with obesity and severe obesity was lower than patients with a BMI in the healthy range (OB = OR 0.80, 95% CI 0.66-0.97 and SO = OR 0.61, 95% CI 0.44-0.85). |

| | | | |
|---|---|---|---|
| | | | The OR and CI became insignificant after adjustment for baseline characteristics and for baseline characteristics and sepsis interventions. |
| Rae et al., 2013, USA | Retrospective analysis of multicentre trial<br>Defined obesity using the NIH/WHO classification | N = 296<br>NW = 106 (35.8%)<br>OW = 95 (32.1%)<br>OB = 80 (27%)<br>SO = 15 (5.1%) | Men outnumbered women among patients with a BMI in the healthy range (82%), overweight (76%), and obesity (74%) but accounted for only 40% of those with severe obesity (p = 0.004).<br>Age (p = 0.84) and APACHE score (p = 0.78) were similar across BMI categories.<br>The incidence of ARDS, multi organ failure, and sepsis were not significantly different across BMI categories.<br>No differences in hospital and ICU length of stay between patients with severe obesity and those in the other groups.<br>Severe obesity was identified as an independent risk factor for mortality in patients with burn injury (OR 10.1, 95% CI 1.94-52.5, p = 0.006). |
| Wang et al., 2013, USA | Retrospective cohort study<br>Defined obesity based on BMI and WC | BMI groups, n= 29,966<br>UW = 319 (1%)<br>NW = 7,091 (23.7%)<br>OW = 11,057 (36.9%)<br>OB = 9,640 (32.2%)<br>SO = 1,859 (6.2%)<br>WC groups, n = 30,183<br>NWC = 15,448 (51.2%)<br>LWC = 14,735 (48.8%) | BMI and WC were higher among younger individuals, females and African Americans.<br>Patients with high BMI and WC were more likely to have chronic diseases, such as chronic lung disease, diabetes, stroke, coronary artery disease, hypertension, and dyslipidaemia.<br>Among the 975 sepsis events, the most common infection types were pneumonia (43.8%), kidney and urinary tract infections (15.9%), and abdominal infections (13.6%).<br>Severe obesity and large WC were independently associated with an increased risk of sepsis (HR 1.57, 95% CI 1.16-2.14 and HR 1.34, 95% CI 1.15-1.56, respectively).<br>When both BMI and WC were included as independent variables in the model, large WC remained independently associated with sepsis (HR 1.47, 95% CI 1.20-1.79). Stratification by BMI revealed that the association between WC and sepsis risk was significant only among patients with overweight (HR 1.42, 95% CI 1.10-1.83) and obesity (HR 1.80, 95% CI 1.12-2.90) groups. |
| Gaulton et al., 2014, USA | Retrospective cohort study<br>Defined obesity using the WHO classification | N = 1,779<br>UW (excluded from analysis) = 126 (7.1 %)<br>NO = 1,080 (60.6 %)<br>OB = 573 (32.2 %) | More than 60% of patients in both groups identified as White.<br>Patients' ages were similar between the BMI groups (p = 0.65).<br>Patients with obesity were more likely to be female and to receive vasopressor than those without obesity (49.4% vs. 36.2%, p < 0.001 and 36.5% vs. 28%, p < 0.001, respectively).<br>Patients with obesity had significantly higher rates of COPD, diabetes, and hypertension but lower rates of leukaemia/lymphoma and transplantation compared to those without obesity.<br>The median LOS following presumed sepsis was comparable between BMI groups (9.53 (4.01-19.7) days vs. 9.52 (3.96-19.9) days, p = 0.45).<br>No significant association between obesity and increased odds of mortality after adjusting for ICU location and administration of a vasopressor (OR 1.11, 95 % CI 0.85-1.41, p = 0.47).<br>There were no significant association between BMI and mortality (p = 0.14).<br>The mortality rates were comparable across BMI categories (p = 0.18).<br>Patients with obesity had non-significantly higher odds of mortality at 3 days and at 7 days (OR 1.36, 95 % CI 0.98-1.89, p = 0.06 and OR 1.22, 95 % CI 0.91-1.62, p = 0.19, respectively). |

| | | | |
|---|---|---|---|
| Mica et al., 2014, Switzerland | Retrospective study<br>Defined obesity based on BMI | N = 651<br>NW = 378 (58.1%)<br>OW = 224 (34.4%)<br>OB = 49 (7.5%) | Men comprised 70% of patients with a BMI in the healthy range, 85% with overweight, and 82% with obesity (p < 0.0001).<br>The mean age was 42.9 (18.4) years and was comparable between BMI groups (p = 0.632).<br>No significant differences in APACHE II score among the groups.<br>Patients with a BMI in the healthy range showed a significantly higher maximum SIRS score (3.4 ± 0.8) than those with overweight (2.3 ± 1.1) and obesity (2.47 ± 1.1).<br>The sepsis incidence was higher among patients with a BMI in the healthy range compared to those with overweight and obesity (46.1% vs. 0.2% vs. 0%, respectively, p < 0.0001).<br>No significant differences in clinical outcomes among the groups, including hospital stay (0.612), ICU stay (0.992), ventilator days (0.898), and mortality (0.157). |
| Gaulton et al., 2015, USA | Retrospective cohort study<br>Defined obesity according to the NIH criteria | N= 1,191<br>UW = 102 (8.6%)<br>NW = 480 (40.3%)<br>OW = 301 (25.3%)<br>OB = 229 (19.2%)<br>SO = 79 (6.6%) | Patients in with severe obesity and obesity were younger (p = 0.04) and less likely to be male (p < 0.001) compared to those with a BMI in the healthy range and underweight.<br>APACHE II and SOFA scores were comparable across the BMI groups.<br>Patients with obesity and severe obesity had significantly higher rates of hypertension, diabetes, and congestive heart failure, and less likely to have a cancer or be immunosuppressed than those without obesity.<br>The combined group of patients with obesity and severe obesity showed a non-significant decreased in mortality risk compared to those with a BMI in the healthy range (OR 0.66, CI 0.42-1.03, p = 0.06). |
| Goode et al., 2016, USA | Correlational study using secondary electronic data<br>Defined obesity using the WHO classification | N = 1,917<br>Patients without postoperative sepsis, n = 1,897 (99%):<br>UW = 32 (1.69%)<br>NW = 421 (22.19%)<br>OW = 580 (30.57%)<br>OB class I = 437 (23.04%)<br>OB class II = 233 (12.28%)<br>OB class III = 194 (10.23%)<br>Patients with postoperative sepsis, n = 20 (1%):<br>UW = 1(5%)<br>NW = 3 (15%) | Of total 30,549 patients, 25,043 (81.98%) were White.<br>Among patients with sepsis, 50.34% were female, compared to 53.49% of patients without sepsis.<br>Patients with post-operative sepsis were younger (53.65 ± 15.69 years) and had more comorbid conditions (2.80 ± 2.14), including CHF, diabetes, hypertension, and metastatic cancer compared to those without sepsis (57.91 ± 15.34 years; 1.78 ± 1.59 comorbidities).<br>Patients with sepsis experienced longer hospital stays (18.96 ± 16.61 days) than those without sepsis (5.38 ± 6.28 days)<br>BMI was a positive predictor of sepsis occurrence (OR 1.05, p = 0.05) when adjusted for length of stay. LOS was also a significant positive predictor (OR 1.11, p = 0.05). |

| | | | |
|---|---|---|---|
| | | OW = 4 (20%)<br>OB class I = 4 (20%)<br>OB class II = 2 (10%)<br>OB class III = 6 (30%) | |
| Palakshappa et al., 2016, USA | Prospective cohort study<br>Defined obesity using the WHO criteria | N = 163<br>Patients without ARDS (n = 90):<br>UW = 11 (12%)<br>NW = 43 (48%)<br>OW = 18 (20%)<br>OB = 18 (20%)<br>Patients with ARDS (n = 73):<br>UW = 8 (11%)<br>NW = 23 (32%)<br>OW = 19 (26%)<br>OB = 23 (32%) | Of all patients, 89 (55%) were White.<br>Patients with ARDS had a higher median BMI compared to those without ARDS, but this was not statistically significant (25.6 (21.3, 31.0) vs. 23.6 (20.3, 28.6), p = 0.15).<br>Compared to patients without ARDS, those with ARDS were more likely to have a history of liver disease (p = 0.003), shock on admission (p = 0.03), a pulmonary source of sepsis (p = 0.002), higher APACHE III score (0.003), and experience higher 30-day mortality (P < 0.001).<br>The median adiponectin concentration was lower among patients with obesity compared to those without obesity (7.08 (2.88-11.43) mcg/ml and 8.84 (4.56-18.53) mcg/ml, respectively). Adiponectin level was inversely associated with BMI (p = 0.01).<br>Patients with ARDS on admission demonstrated higher plasma adiponectin levels than those without ARDS (OR 1.11, 95 % CI 1.00-1.23, p = 0.048), independent of BMI.<br>There was no significant association between adiponectin and mortality in the complete adjusted multivariable model (OR 1.02, 95 % CI 0.92-1.15, p = 0.66). |
| Papadimitriou Olivgeris et al., 2016, Greece | Retrospective cohort study<br>Defined obesity using the WHO criteria | N = 834<br>NO = 671 (80.5%)<br>OB = 163 (19.5%), including 25 (3%) patients with SO | Patients with obesity were more likely to be female (43.6%) compared to those without obesity (32.2%) (p = 0.035).<br>The patients' ages were comparable between the BMI categories (p = 0.095).<br>Patients with obesity had significantly higher rates of diabetes and COPD than those without obesity.<br>No significant differences in APACHE II (p = 0.679), SAPS II (p = 0.412), and SOFA (0.051) scores at admission across the BMI categories.<br>Patients with obesity stayed longer in the ICU (18.4 ± 9.7 days) than those without obesity (8.8 ± 11.4 days) (p = 0.004).<br>Patients with obesity had increased ICU mortality (28.8%) compared to those without obesity (21%) (p = 0.036).<br>Sepsis was the primary condition of admission and was associated with lower survival in patients with obesity compared to those without (76.3% vs. 43.7%, p = 0.001).<br>Multivariate analysis identified that obesity (OR 5.3, 95% CI 1.4-20.2, p = 0.014) and development of septic shock (OR 3.4, 95% CI 1.3-9.1, p = 0.015) were independently associated with mortality.<br>Kaplan-Meier curves revealed that the 30-day survival probability was lower among patients with obesity compared with those without obesity. |

| | | | |
|---|---|---|---|
| Parker et al., 2016, USA | Retrospective cohort study<br>Defined obesity based on BMI | N = 3,187<br>Patients without infection, n = 2,421 (76%):<br>NO = 1,630 (67.9%)<br>OB (770; 32.1%)<br>Patients with infection = 766 (24%):<br>NO = 439 (57.5%)<br>OB = 324 (42.5%) | The median age of patients was 70 (62-77) years, with 81.8 male and 82.8% Non-Hispanic White.<br>Among patients with infections, 404 (12.7%) had surgical site infection, 405 (12.7%) had sepsis/septic shock, and 309 (9.7%) experienced urinary tract infection.<br>Patients suffering an infection had a longer LOS (9 (7-16) days) than patients without infection (7 (6-10) days) ($p < 0.001$).<br>Postoperative infections were more common among patients with obesity than those without obesity (42.5% vs. 32.1%, $p < 0.01$).<br>Obesity was independently associated with an increased risk of infection (OR 1.52, 95% CI 1.27–1.81, $p < 0.01$) and sepsis/septic shock (OR 1.39, 95% CI 1.11–1.74, $p < 0.01$) within 30 days of surgery. |
| Wong et al., 2017, USA | Retrospective cohort study<br>Defined obesity using the NHLBI classification | N = 33<br>NW = 20 (60.6%)<br>SO = 13 (39.4%) | Baseline characteristics including age, sex (men), comorbidities, and predicted mortality were similar between groups.<br>Average weight-based dosing of norepinephrine was comparable between patients with a BMI in the healthy range and those with severe obesity on days one (0.19 vs. 0.14 mcg/kg/min, p= 0.151) and two (0.13 vs. 0.08 mcg/kg/min, p= 0.136).<br>No significant difference in the time to goal MAP between the BMI groups (1.95 vs. 2.46 h, p = 0.511).<br>No significant difference between groups in the incidence of tachycardia on days one and two after norepinephrine administration.<br>Hospital LOS, ICU LOS, and mortality rates were similar between groups (p = 0.312, p = 0.468, and p = 0.930, respectively). |
| Gameiro et al., 2018, Portugal | Retrospective cohort study<br>Defined obesity based on BMI | N = 456<br>NO = 331 (72.6%)<br>OB = 125 (27.4%) | 433 (94.9%) patients were White.<br>No significant difference in age between the BMI groups (p = 0.779).<br>Patients without obesity were more likely to be male than those with obesity (61.3% vs. 48.8%, p = 0.016).<br>Compared to patients without obesity, those with obesity had higher rates of diabetes (32% vs. 19%, p = 0.003) and hypertension (56.8% vs. 43%, p = 0.007), but had lower rates of neoplasia (12.8% vs. 28.1%, p = 0.001) and respiratory infection (21.6% vs. 33.5%, p = 0.013).<br>Patients with obesity had higher SAPS II scores (52.1 ± 17.1 vs. 48.4 ± 17.3, p = 0.43) and higher haemoglobin concentrations (10.8 ± 2 vs. 10.2 ± 2, p = 0.005) at ICU admission compared to those without obesity.<br>Patients with obesity developed AKI more frequently than those without obesity (OR 2.31, 95% CI 1.07–5.02, p= 0.034); obesity was associated with AKI (OR 2.3, 95% CI 0.011–0.146, p= 0.024). Patients with obesity were more likely to require renal replacement therapy than those without obesity (37.9% vs. 22.6%, $p < 0.001$).<br>There was no significant association between obesity and mortality, even after excluding patients with underweight (unadjusted OR 0.4, 95% CI 0.12-0.08, p = 0.71). |

| | | | |
|---|---|---|---|
| Ji et al., 2018, China | Retrospective cohort study<br>Defined obesity using body composition parameters | N = 236<br>No SR or VO = 64 (27.1%)<br>VO = 58 (24.6%)<br>SR = 62 (26.3%)<br>SRO = 52 (22%) | Patients with sarcopenia and sarcopenic obesity were older (76 (65-81) years and 75 (69–83) years, respectively) than those with visceral obesity (66 (53-74) years) and those without sarcopenia and visceral obesity (58 (44-69) years).<br>Patients with sarcopenia had a higher proportion of females (51.6%) compared to patients with sarcopenic obesity (42.3%), visceral obesity (27.6%), and those without either condition (42.2%).<br>Patients with sarcopenic obesity had higher APACHE II and SOFA scores compared to those in the other groups.<br>Thirty-day mortality differed significantly among the groups ($p < 0.001$), with patients with sarcopenic obesity having the highest mortality rate (44.2%) compared to those with sarcopenia (29%), visceral obesity (19%) and neither condition (7.8%).<br>Patients with sarcopenic obesity had a longer ICU stay (6 (3-14) days) than those with sarcopenia (5 (3-10) days), visceral obesity (5 (2-10) days), and neither condition (3 (2-5) days) group ($p < 0.001$).<br>Sarcopenic obesity was the only body composition parameter independently associated with an increased risk of 30-day mortality (model 1: HR 4.6, 95% CI 1.7-12.8, p = 0.003; model 2: HR 4.2, 95% CI 1.5-11.6, p = 0.007). |
| Katayama et al., 2018, Japan | Retrospective cohort study<br>Defined obesity based on BMI | N = 569<br>Patients diagnosed with AKI, actual body weight (ABW) group = 53 (26.9%):<br>UW = 12 (7.8%)<br>NW = 83 (54.2%)<br>OW = 40 (26.1%)<br>OB = 18 (11.8%)<br>Patients diagnosed with AKI, ideal body weight (IBW) group = 140 (24.6%):<br>UW = 19 (13.6%)<br>NW = 80 (51.2%)<br>OW = 28 (20%)<br>OB = 13 (9.3%) | Patients with obesity were younger in both the ABW and IBW groups (p = 0.006 and p = 0.0015, respectively).<br>No significant differences in sex (male) across the BMI subgroups in both the ABW and IBW groups.<br>Although illness severity (SAPS II and SOFA scores) was similar among BMI subgroups, patients with underweight had higher mortality rates compared to other subgroups in the ABW group (UW 76.7% vs. NW 39.5% vs. OW 26.0% vs. OB 35.7%, p= 0.033).<br>The mortality rate was not significantly different among BMI subgroups in the IBW group (p = 0.736).<br>Using ABW to calculate weight-adjusted hourly urine output for AKI diagnosis increased sensitivity compared to IBW, regardless of disease severity in both groups. |

| | | | |
|---|---|---|---|
| Koch et al., 2018, Germany | Prospective cohort study<br>Defined obesity based on BMI | N = 229 (Patients with sepsis = 142 (62%), patients without sepsis, n = 87 (38%))<br>NO = 158 (76%)<br>OB = 50 (24%) | Of the patients, 133 (58%) were male, the median age was 63 (18-90) years. 32% of them had preexisting diabetes and 10% had preexisting cirrhosis.<br>Patients with sepsis had higher APACHE II score (18 (3-43)) than those without sepsis (14.5 (2-33)).<br>Visfatin serum concentrations were associated with sepsis (p = 0.040), and illness severity (APACHE II > 10, p = 0.001) but not with obesity and type 2 diabetes at ICU admission.<br>Increased visfatin levels at ICU admission predicted the overall mortality during a two-year follow-up period. |
| Kok et al., 2018, Canada | Retrospective cohort study<br>Defined obesity according to the WHO classification | N = 362<br>UW = 21 (5.8%)<br>NW = 132 (36.5%)<br>OW = 92 (25.4%)<br>OB = 83 (22.9%)<br>SO = 34 (9.4%) | Patients with severe obesity were slightly older (58.3 ± 10.1 years) than those with a BMI in the healthy range (57.7 ± 13.1 years).<br>Patients with obesity and severe obesity had a higher proportion of females (41% and 41.2%, respectively) than those with a BMI in the healthy range (29.5%).<br>Patients with severe obesity had highest rates of cardiac illness (38.2%), diabetes (41.2%), and chronic lung disease (26.5%) among all groups.<br>Median Charlson comorbidity index scores increased with higher BMI categories.<br>MELD scores were higher among patients with overweight (36.5 ± 10.2), obesity (38.5 ± 9.8) and severe obesity (37.9 ± 10.9) compared to those with a BMI in the healthy range (33.6 ± 10.4).<br>Compared to patients with a BMI in the healthy range (27.02 ± 7.96), APACHE II scores were higher in patients with obesity (27.63 ± 7.87) but lower in those with severe obesity (25.12 ± 7.72).<br>ICU LOS was longer in patients with overweight (8.0 (4.0-14.0) days), obesity (8.0 (4.0-16.0) days) and severe obesity (7.5 (4.0-13.2)) than in those with a BMI in the healthy range (7.0 (4.0-14.0) days).<br>Compared to patients with a BMI in the healthy range, hospital and 90-day survivals were decreased among those with overweight, obesity and severe obesity.<br>BMI was independently associated with increased in-hospital mortality (OR 1.07, 95% CI 1.005-1.129, p = 0.034). |
| Lee et al., 2018, Canada | Retrospective cohort study<br>Defined obesity based on the ratio of visceral adipose tissue (VAT) to subcutaneous adipose tissue (SAT) | N = 75<br>Low VAT/SAT = 36<br>High VAT/SAT = 37 | The majority of patients were Caucasian, accounting for 93.9% in patients with low VAT/SAT and 80% in those with high VAT/SAT.<br>Compared to patients with low VAT/SAT, those with high VAT/SAT were older (p = 0.025), predominantly male (p < 0.001), had higher APACHE II scores (p = 0.028), and higher creatinine levels at admission (p = 0.039).<br>No significant differences in chronic conditions between the groups.<br>Patients with a low VAT/SAT ratio demonstrated higher 90-day survival rates (p = 0.039) and higher LDL levels (p = 0.024) than those with a high VAT/SAT.<br>Among patients not on statin therapy, LDL levels were higher among patients with low VAT/SAT compared to those with high VAT/SAT (p = 0.006). The higher LDL levels among patients with low VAT/SAT were associated with improved 90-day survival (p = 0.019). |

| | | | |
|---|---|---|---|
| Taylor et al., 2018, USA | Retrospective study<br>Defined obesity based on the WHO classification | N= 4,126<br>UW = 315 (7.6%)<br>NW = 1,401 (34%)<br>OW = 1,117 (27%)<br>OB = 925 (22.4%)<br>SO = 368 (8.9%) | Higher BMI was associated with younger age ($p < 0.001$) and a greater proportion of males ($p < 0.001$).<br>Patients with obesity and severe obesity had significantly higher frequency of CHF, diabetes, and cirrhosis, but lower rate of malignancy and dementia compared to those with a BMI in the healthy range or underweight.<br>Patients with higher BMI received significantly less fluid per kilogram (ml/kg actual body weight) at 3 hour ($p < 0.0001$) and 6 hour ($p < 0.0001$) than patients with lower BMI.<br>Patients with severe obesity had lower hospital and ICU mortality ($p = 0.026$ and $p = 0.001$, respectively) compared to those in other BMI categories.<br>In the adjusted analysis, BMI grouping was not an independent predictor of mortality, with the AUC was 0.79. |
| Tsolakoglou et al., 2020, Greece | Prospective observational study<br>Defined obesity based on BMI | N = 744<br>UW = 52 (6.9%)<br>NW = 348 (46.8%)<br>OW = 256 (34.4%)<br>OB = 72 (9.7%)<br>SO = 16 (2.2%) | The mean age of patients was 63.6 ± 16.6 years.<br>376 (50.5%) of patients were male.<br>The average APACHE II and MODS scores were 23.3 ± 6.9 and 7.5 ± 3.8, respectively.<br>Among 722 patients with central venous catheters, 178 (24.7%) developed Central-Line-Associated Bloodstream Infections (CLABSIs).<br>Patients with CLABSIs had a greater proportion of patients with overweight and obesity (78.7%) compared to those without CLABSIs (49%).<br>The frequency of diabetes was higher among patients with CLABSIs (22.9%) compared to those without CLABSIs (19.3%) ($p = 0.034$).<br>Patients with CLABSIs had significantly higher MODS scores, longer ICU and hospital LOS, and a higher mortality rate than those without CLABSIs.<br>BMI was a significant predictor of CLABSIs, with higher BMI associated with increased risk (OR 1.223, 95% CI 1.107-1.351, $p = 0.001$). |
| Weber et al., 2020, USA | Retrospective cohort study<br>Defined severe obesity based on BMI | N = 2,019<br>Before propensity matching:<br>NO = 1,606 (79.5%)<br>SO = 413 (20.5%)<br>After matching:<br>NO = 397 (50%)<br>SO = 397 (50%) | The majority of patients were White, accounting for 87.9% of those with obesity and 87.4% of those without obesity.<br>Post propensity score matching, age, sex, and chronic disease rates were balanced between patients with severe obesity and those without obesity, as indicated by a maximum absolute standardised difference of 0.041.<br>No significant differences in LOS and postoperative sepsis were observed between the BMI groups, both before and after propensity matching.<br>Before matching, the incidence of postoperative septic shock was significantly higher among patients with severe obesity than those without obesity (18% vs. 12%, $p = 0.004$); this difference was no longer significant after propensity matching (16.6% vs. 15.4%, $p = 0.699$). |

| | | | |
|---|---|---|---|
| Winter-Jensen et al., 2020, Denmark | Mendelian randomisation study<br>Defined obesity based on BMI | N = 101,447<br>UW = 824 (0.8%)<br>NW = 44,107 (43%)<br>OW = 40,616 (40%)<br>OB = 15,900 (16%) | Individuals with overweight (59 ± 13 years) and obesity (60 ± 12 years) were older compared to those with a BMI in the healthy range (56 ± 13 years).<br>A lower proportion of women was observed in patients with overweight (40%) and obesity (50%) when compared to those with a BMI in the healthy range (65%).<br>Individuals with obesity had the highest percentage of type 2 diabetes (13%) followed by those with overweight (5%), a BMI in the healthy range (2%), and underweight (1%).<br>Over a follow-up period of 8.8 years, 10,263 individuals (12%) had hospital contact for an infection. This included 5,194 cases (5%) of pneumonia, 2,511 (3%) of skin infections, 2,930 (3%) of urinary tract infections, and 2,394 (2%) of sepsis.<br>Compared to individuals with a BMI in the healthy range, those with obesity had increased risk of any infection (HR 1.37, 95% CI 1.30-1.45), pneumonia (HR 1.20, 95% CI 1.11-1.30), skin infection (HR 2.02, 95% CI 1.81-2.24), urinary tract infection (HR 1.46, 95% CI 1.32-1.62), and sepsis (HR 1.47, 95% CI 1.32-1.65).<br>Using a genetic risk score as an instrumental variable for BMI, genetically induced higher BMI was associated with increased odds of skin infection (OR 1.12, 95% CI 1.03-1.22 per genetically induced 1-unit increase in BMI). |
| Abumayyaleh et al., 2021, Germany | Retrospective cohort study<br>Defined obesity according to the WHO criteria | N = 3,635<br>BMI < 25 $kg/m^2$, n = 1,110 (30.5%)<br>OW = 1,464 (40.3%)<br>OB = 1,061 (29.2%) | Patients with obesity were more likely to be male compared to those with BMI < 25 $kg/m^2$ (29% vs. 24.9%, p < 0.001).<br>Patients with obesity were older than those with BMI < 25 $kg/m^2$ (< 70 years: 27% vs. 32.6%).<br>Arterial hypertension, dyslipidaemia, diabetes, and renal insufficiency were significantly higher among patients with obesity compared to those with BMI < 25 $kg/m^2$.<br>The incidence of sepsis was significantly higher in patients with obesity than in those with BMI < 25 $kg/m^2$ (32.5% vs. 25.1%, p = 0.009).<br>Mortality was higher among patients with obesity compared to those with BMI < 25 $kg/m^2$ (33.5% vs. 27.2%, p = 0.004).<br>Neither a BMI < 25 $kg/m^2$ nor a BMI > 30 $kg/m^2$ significantly impacted mortality (HR 1.15, 95% CI 0.889-1.508, p = 0.27; and HR 1.15, 95% CI 0.893-1.479, p = 0.27, respectively). |
| Butler-Laporte et al., 2021, UK | Mendelian randomisation study<br>Defined obesity using BMI | 698 BMI-related single-nucleotide polymorphisms | Increasing BMI was associated with higher rates of admissions due to all infectious diseases, including sepsis (OR 1.05, 95% CI 1.03-1.07) with the largest effect in soft tissue infections (SSTIs) (OR 1.11, 95% CI 1.09-1.12).<br>Higher BMI was associated with increased 28-day sepsis mortality (OR 1.08, 95% CI 1.02-1.13) and pneumonia mortality (OR 1.03, 95% CI 1.01-1.05).<br>Mendelian randomisation-Egger analyses indicated small pleiotropic effects for admissions due to all infections (α $5.2 \times 10^{-4}$, CI $1.9 \times 10^{-4}$, $8.6 \times 10^{-4}$) and pneumonia admissions (α $6.2 \times 10^{-4}$, CI $7.0 \times 10^{-5}$, $1.2 \times 10^{-3}$). |

| | | | |
|---|---|---|---|
| | | | Mendelian randomisation-Steiger analyses confirmed that increased BMI likely contributed to higher admission rates for pneumonia, urinary tract infections (UTIs), SSTIs, sepsis, and all infectious diseases (the causal direction: $p < 5 \times 10^{-7}$). |
| Ozben et al.**,** 2021, Türkiye | Retrospective cohort study<br>Defined obesity using the WHO criteria | N = 147<br>NO = 105 (71.4%)<br>OB = 42 (28.6%) | Patients characteristics including age, sex, comorbidities, ASA scores, site of tumour, and neoadjuvant chemotherapy use were similar between BMI groups.<br>No significant difference in intraoperative complications between patients without and with obesity (3.8% vs. 0%, p > 0.99).<br>No significant difference in postoperative outcomes between groups, including sepsis, hospital stays, and mortality (p > 0.99). |
| Page-Wilson et al.**,** 2021, USA | Retrospective cohort study<br>Defined obesity using BMI | N = 1,019<br>UW = 25 (2.5%)<br>NW = 227 (22.3%)<br>OW = 347 (34%)<br>OB = 340 (33.3%)<br>SO = 80 (7.9%) | The racial composition of the study population was as follows: 23.9% White, 23% Black, 2.1% Asian, 34.2% Other, and 16.8% Not Specified; no significant differences across BMI groups.<br>Compared to patients with a BMI in the healthy range (71.0 (59.0–81.5) years), those with overweight (60.0 (50.0–71.0)), obesity (65.0 (54.0–75.5)), and severe obesity (52.0 (40.0–65.3)) were younger (p < 0.001).<br>Patients with obesity and severe obesity had less proportion of males (50.9% and 51.2%, respectively) than those with a BMI in the healthy range (56.4%) (p < 0.001).<br>Patients with obesity and severe obesity had significantly higher rates of asthma and obstructive sleep apnoea, but lower proportion of active cancer and renal disease compared to those with a BMI in the healthy range.<br>Over the study period a total of 22.7% patients developed septic shock and 23.7% patients died.<br>Increasing BMI was independently associated with a higher risk of death (OR 1.04, CI 1.01–1.06), with BMI ≥ 40kg/m2 had the highest odds of mortality (OR 2.05, CI 1.04–4.04).<br>BMI as a continuous variable was an independent factor for septic shock (OR 1.04, 95% CI 1.01–1.06),<br>Patients with obesity (OR 1.88, 95% CI 1.18–2.97) and severe obesity (OR 2.38, 95% CI 1.26–4.51) had significantly higher odds of developing septic shock compared to those with a BMI in the healthy range. |
| Lameka et al.**,** 2022, USA | Case-control study<br>Defined obesity based on BMI, according to the WHO, the NIH, and the NHLBI definition of obesity | N = 428<br>NW = 109 (25.4%)<br>OW =133 (31.1%)<br>OB = 133 (31.1%)<br>SO = 53 (12.4%) | Patients with obesity (44 years) and overweight (43 years) were older than those with severe obesity (37 years) and a BMI in the healthy range (38 years) (p = 0.0076).<br>Patients with severe obesity had a fewer proportion of male patients (41.5%) compared to those with obesity (69.17%), overweight (75.19%), and a BMI in the healthy range (72.4%) (p < 0.001).<br>Patients with obesity and severe obesity had longer LOS (12.8 and 11 days, respectively) than those with overweight (9.6 days) and a BMI in the healthy range (8.9 days) (p = 0.0153).<br>No significant differences were observed in comorbidities across BMI categories.<br>There was no significant difference in sepsis occurrence between groups (p = 0.3210). |

| | | | |
|---|---|---|---|
| | | | When comparing patients with severe obesity with all other BMI groups, no significant differences were found in the rates of systemic complications, overall incidence of all-cause complications, LOS, and mortality. |
| Lee et al., 2022, Taiwan | Retrospective database study<br>Defined obesity using ICD-9-CM codes | N = 23,898<br>NO = 21,069 (88.2%)<br>OB = 1,812 (7.6%)<br>SO = 1,017 (4.2%) | Patients with severe obesity (62.34 ± 11.26 years) and obesity (64.43 ± 11.70 years) were younger than those without obesity (67.76 ± 13.26 years, $p < 0.0001$).<br>The majority of patients (75.83%) were White; patients with obesity had the lowest proportion of White race as compared to the other two BMI groups ($p < 0.001$).<br>51.18% of patients were females, with no significant differences in sex distribution across the groups.<br>Patients with severe obesity had the highest rates of comorbidities, including CHF ($p < 0.0001$), chronic pulmonary disease ($p < 0.0001$), coagulopathy ($p = 0.0268$), diabetes ($p < 0.0001$), liver disease ($p < 0.0001$), fluid/electrolyte disorders ($p = 0.0119$), and renal failure ($p < 0.0001$).<br>Patients with severe obesity had the highest proportion of postoperative complications involving infection/sepsis (NO 2.84%; OB 2.43%; SO 4.13%, $p = 0.0280$), respiratory (NO 5.47%; OB 6.13%; SO 8.65%, $p < 0.001$), AKI (NO 4.70%; OB 5.08%; SO 8.75%, $p < 0.001$), and DVT/PE (NO 0.68%; OB 1.21%; SO 1.67%, $p = 0.0001$).<br>Severe obesity was independently associated with increased risk of in-hospital mortality (OR 2.06, 95% CI 1.11-3.83), AKI (OR 1.78, 95% CI 1.34-2.36), deep vein thrombosis/pulmonary embolisms (OR 2.88, 95% CI 1.70-4.88) and extended LOS (OR 1.21, 95% CI 1.02-1.43).<br>Obesity was significantly and independently associated with increased risk of deep vein thrombosis/pulmonary embolisms (OR 2.12, 95% CI 1.32-3.41). |
| Lenney et al., 2022, USA | Retrospective chart review study<br>Defined obesity using the CDC criteria | N = 219<br>BMI <25 kg/m$^2$ = 56 (25.5%)<br>OW = 54 (24.7%)<br>OB = 54 (24.7%)<br>SO = 55 (25.1%) | Baseline characteristics, including age, sex (male), and SOFA score were comparable between groups.<br>Compared to patients without obesity, patients with obesity and severe obesity had a significantly higher proportion of patients with a past medical history of coronary artery disease and diabetes.<br>History of ESRD ($p = 0.04$) and immunocompromise ($p = 0.00$) was more common in patients with obesity than in those with severe obesity, overweight, or BMI < 25.<br>The mean norepinephrine dose and mean MAP were similar across BMI categories at 6, 12, and 24 hours.<br>No significant differences among the BMI groups for secondary outcomes, including hospital LOS ($p = 0.41$), ICU LOS ($p = 0.79$), hospital mortality ($p = 0.30$), and ICU mortality ($p = 0.36$). |
| Tay-Lasso et al., 2022, USA | Retrospective cohort study<br>Defined obesity using BMI | N = 1,246<br>NO = 566 (45.4%)<br>OB = 680 (54.6%) | The majority of patients were White, 74.5% of those with obesity and 73.1% of those without obesity.<br>Patients with obesity were more likely to be male than those without obesity (73.8% vs. 68.6%, $p < 0.001$).<br>The median age of patients with obesity was lower compared to those without obesity (54 (60-80) years and 60 (64-85), respectively, $p < 0.001$). |

| | | | |
|---|---|---|---|
| | | | Patients with obesity had higher rates of hypertension (55.1% vs. 42.6%, p < 0.001), chronic renal failure (7.1% vs. 5.8%, p < 0.001), and diabetes but a lower rate of COPD (14.6% vs. 17.7%, p < 0.001) than those without obesity.<br>Patients with obesity had an increased hospital LOS and ICU LOS than those without obesity (22.0 (12.0-36.0) days vs. 19.0 (10.25-32.0) days, p < 0.001 and 14.0 (7.0-25.0) vs. 11.0 (5.0-20.0), p < 0.001, respectively).<br>The risk of mortality was similar among BMI categories. |
| Tolley et al. 2022, USA | Retrospective cohort study<br>Defined obesity using the WHO classification | N = 37,502. Of those patients, 7,243 patients underwent at least one burn surgery were evaluated for operation outcomes and complications, including sepsis. The patients by BMI class, n = 7,507, as follows:<br>UW = 200 (2.7%)<br>NW = 2,736 (36.4%)<br>OW = 2,334 (31.1%)<br>OB class I = 1,313 (17.5%)<br>OB class II = 548 (7.3%)<br>OB class III = 376 (5%) | Of all patients, 70.5% were male, with a mean age of 45.7 years (SD 17.2) and a median BMI of 26.9 (23.4-31.3). The mean total body surface area affected in those undergoing surgery was 17.3% (SD 13.0).<br>Patients with underweight (OR 1.13, 95% CI 1.01–1.26, p = 0.032) and those with Class III obesity (OR 1.19, 95% CI 1.04–2.29, p = 0.042) had higher odds of death compared to patients with a BMI in the healthy range.<br>Patients with underweight had the highest rate of sepsis (11; 5.3%) amongst the other BMI groups: NW (49; 2.0%), OW (39; 1.6%), OB class 1 (46; 3.4%), OB class II (23; 4.0%); OB class III (10, 2.5%).<br>Class II obesity and underweight were associated with increased odds of sepsis (OR 2.46, 95% CI 1.43-4.27, p= 0.001 and OR 3.35, 95% CI 1.62-6.94, p = 0.001, respectively).<br>Class III obesity was associated with increased odds of renal (OR 2.10, 95% CI 1.17–3.76, p = 0.013) and cardiac (OR 3.89, 95% CI 2.02 to 7.45, P < 0.001) complications.<br>Class III obesity was associated with longer ICU stay (coefficient 1.98, 95% CI 0.52-3.45, p = 0.008) and increased mortality (OR 1.19, 95% CI 1.04-2.29, p = 0.042). |
| Ward et al. 2022, USA | Retrospective cohort study<br>Defined obesity using the WHO categories | N= 1,032<br>UW = 105 (10.2%)<br>NW – OW = 631 (61.1%)<br>OB = 296 (28.7%) | Patients with obesity were younger (p < 0.001) and more likely to be female (p = 0.001).<br>Patients with obesity had a significantly lower history of heart failure and a lower rate of septic shock than those in other BMI groups.<br>30by3 dosing based on actual versus ideal body weight was achieved in 80.0% vs. 52.4% of patients with underweight, 56.4% vs. 56.9% of those with a BMI in the healthy to overweight range, and 23.3% vs. 46.0% of patients with obesity.<br>Patients with obesity who did not receive 30by3 dosing had longer ICU stays with both ABW-based (ß = 3.33, 95% CI 0.25–6.41) and IBW-based fluid dosing (ß = 3.22, 95% CI 0.27–6.17).<br>Not receiving 30by3 was associated with increased mortality for patients with underweight, for both ABW (OR 5.82, 95% CI 1.32-25.57) or IBW (OR 2.41, 95% CI 0.64-9.09). |

| | | | |
|---|---|---|---|
| Yildiz et al. 2022, Türkiye | Retrospective cohort study<br>Defined obesity based on BMI | N = 128<br>NW = 46 (35.9%)<br>OB = 42 (32.8%)<br>SO = 40 (31.3%) | The mean age was 57.2 ± 9.8 years, with no significant differences between groups (p = 0.242).<br>Compared to patients with a BMI in the healthy range, those with obesity or severe obesity had longer hospital stays (p = 0.029) and an increased risk of postoperative complication (p = 0.004).<br>No significant difference in sepsis events across the BMI categories (p = 0.312).<br>Severe obesity (HR = 6.03, p = 0.008) and ICU admission (HR = 10.2, p = 0.001) were identified as independent risk factors for complications.<br>BMI level was positively correlated with the length of hospital stay (r = 0.336, p = 0.007). |
| Colbran et al. 2023, Australia | Retrospective cohort study<br>Defined obesity using the WHO classification | N = 207<br>UW = 7 (3%)<br>NW = 70 (34%)<br>OW =79 (38%)<br>OB = 51 (25%):<br>OB class I (35; 17%)<br>OB class II (10; 5%)<br>OB class III (6; 3%) | Patients with obesity (86%) and overweight (87%) were more likely to be male than those with a BMI in the healthy range (69%) and underweight (43%) (p = 0.002).<br>The age distribution was comparable among groups (p = 0.668).<br>Patients with overweight and obesity were more likely to have diabetes compared to those with a BMI in the healthy range (13% and 10% vs. 0%, respectively, p = 0.007).<br>Patients with obesity were more likely to have CKD (6%, p = 0.035) than those in other BMI categories.<br>Patients with obesity were significantly required a higher rate of admission to the ICU and a longer stay in the ICU than those with a BMI in the healthy range.<br>No significant differences in mortality (p = 0.841) and sepsis incidence (p = 0.721) among groups.<br>The incidence of sepsis was significantly higher among patients with Class 3 obesity (50%) than those with Class 2 (0%) or Class 1 obesity (3%) (p = 0.007). |
| Ning et al. 2023, Germany | Prospective cohort study<br>Defined obesity using The WHO criteria | N = 235<br>Patients without diabetes = 154 (65.5%)<br>NO = 107 (69.5%)<br>OB = 47 (30.5%)<br>Patients with diabetes = 81 (34.5%):<br>NO = 36 (44.5%)<br>OB = 45 (55.5%) | Among patients without diabetes, the median age was 63 years (53-76) for those without obesity and 61 years (54-71) for those with obesity. Among patients with diabetes, the median age was 73 years (63-78) for patients without obesity and 66 years (54-74) for those with obesity.<br>Among patients without diabetes, males accounted for 68% of those without obesity and 70% of those with obesity. Among patients with diabetes, males constituted 83% of those without obesity and 53% of those with obesity.<br>Among patients without diabetes, 27 had sepsis (16 patients without obesity and 11 with obesity) and 11 developed septic shock (10 patients without obesity and 1 with obesity).<br>Among patients with diabetes, 22 patients experienced sepsis (9 patients without obesity and 13 with obesity) and 15 suffered from septic shock (4 patients without obesity and 11 with obesity).<br>Among patients with diabetes, the incidence of septic shock was lower in those without obesity compared to those with obesity (4 vs. 13 cases, respectively, p > 0.05).<br>The frequency of septic shock was higher among patients with both diabetes and obesity compared to those with obesity but without diabetes (11 vs. 1 cases, p < 0.05).<br>The levels of circulating monocytes, CD14+, CD33+, CD14+CD16−, and CD14−CD16+ were significantly decreased (p < 0.05) in patients who developed sepsis and septic shock.<br>The progression from non-sepsis to sepsis and septic shock was associated with significant changes in circulating CD14-positive monocyte levels, independent of diabetes and obesity. |

| | | | |
|---|---|---|---|
| Nooijer et al. 2023, Greece | Secondary analysis of prospective cohort study<br>Defined obesity using the WHO criteria | N= 167<br>UW (excluded from analysis) =2 (1.2%)<br>NW = 67 (40.1%)<br>OW = 56 (33.5%)<br>OB = 42 (25.2%) | Patients with overweight and obesity were younger than patients with a BMI in the healthy range (75 (62–81) and 67 (61–78) years vs. 82 (70–88) years, p = 0.003 and p < 0.001 between subgroups).<br>No significant difference in sex distribution among the groups.<br>Patients with obesity had a higher SOFA score (12 (10–15) vs. 10 (6–12), p < 0.001), coronary heart disease (26% vs. 10%, p = 0.03), and incidence of septic shock septic shock (88% vs. 55%, p < 0.001) than those with a BMI in the healthy range.<br>APACHE II score was comparable across groups (p = 0.16).<br>Patients with obesity had leptin plasma concentrations 2.3-fold higher than patients with a BMI in the healthy range and overweight. There was a positive correlation between leptin and BMI (r = 0.25, p = 0.005).<br>There was no significant difference in leptin concentrations between survivors and non-survivors (AUC of 0.56 [95% CI 0.47-0.65, p = 0.16]).<br>There was no significant difference in 28-day mortality among patients with a BMI in the healthy range, overweight, or obesity (57%, 55%, and 60%, respectively; p = 0.92). |
| **Systematic review studies reported mixed findings on the obesity paradox** | | | |
| Trivedi et al., 2015, USA | Systematic review<br>Defined obesity using the WHO criteria in four studies, the NIH criteria in two studies, and the ICD-9 coding in three studies | N = 9 retrospective cohort studies | Three studies found that patients with obesity or severe obesity was associated with reduced mortality from sepsis compared to those without obesity. One study showed the association of obesity and lower 1-year mortality after sepsis.<br>Five studies reported either inconclusive or conflicting evidence, that there was no association between obesity and reduced short-term sepsis mortality. Of these, two studies initially suggested a protective effect of obesity, but this became insignificant after adjusting for confounders. The three studies had methodological limitations: one investigated presumed sepsis, one was potentially underpowered in the severe obesity category, and one was conducted in a region with higher rates of multidrug-resistant pathogens. |
| Robinson et al., 2020, USA | Systematic review<br>Defined obesity using either prespecified BMI categories or the WHO criteria<br>Defined sepsis, severe sepsis or septic shock with ICD-9 codes | N = 7 studies (6 retrospective cohort studies and 1 prospective cohort study) | Three studies observed significantly decreased mortality in patients with overweight or obesity than patients with a BMI in the healthy range.<br>Three studies found insignificant association between obesity and mortality.<br>One study found a significant positive association between obesity and mortality in patients with bacteraemia. |

AKI, acute kidney injury; AIDS, acquired immunodeficiency syndrome; APACHE, acute physiology and chronic health evaluation; AKI, acute kidney injury; ARDS, acute respiratory distress syndrome; AUC, area under the curve; BMI, body mass index; UW, patients with underweight; NW, patients with a BMI in the healthy range; OW, patients with overweight; OB, patients with obesity; SO, patients with severe obesity; NO, patients without obesity; WC, waist circumference; NWC, patients with a WC in the healthy range, LWC, patients with large WC; SR, patients with sarcopenia; SRO, patients with sarcopenic obesity; VO, patients with visceral obesity; ABW, actual body weight; IBW, ideal body weight; VAT/SAT, visceral adipose tissue/subcutaneous adipose tissue; CAD, coronary artery disease; CHF, congestive health failure; COPD, chronic obstructive pulmonary disease; CRF, chronic renal failure; CCI, Charlson comorbidity index; ESRD, end-stage renal disease; HR, hazard ratio; ICD-9-CM, the International Classification of Diseases Clinical Modification $9^{th}$ Revision; ICU, intensive care unit; LOS, length of stay; LDL, low density lipoprotein; MAP, mean arterial pressure; MELD, model for end-stage liver disease; MODS, multiple organ dysfunction syndrome; NHLBI, the National Heart, Lung, and Blood Institute; NIH, the National Institutes of Health; NYHA, New York Heart Association; OR, odds ratio; SOFA, sequential organ failure assessment score; VLDLR, very low density lipoprotein receptors; WC, waist circumference; WHO, the World Health Organization; WHR, waist-to-hip ratio.
